# Intratumoral Restoration of miR-137 Plus Cholesterol Favors Homeostasis of the miR-137/Coactivator p160/AR Axis and Negatively Modulates Tumor Progression in Advanced Prostate Cancer

**DOI:** 10.3390/ijms24119633

**Published:** 2023-06-01

**Authors:** Ruan Pimenta, Carolina Mie Mioshi, Guilherme L. Gonçalves, Patrícia Candido, Juliana A. Camargo, Vanessa R. Guimarães, Caroline Chiovatto, Vitória Ghazarian, Poliana Romão, Karina Serafim da Silva, Gabriel A. dos Santos, Iran A. Silva, Miguel Srougi, William C. Nahas, Kátia R. Leite, Nayara I. Viana, Sabrina T. Reis

**Affiliations:** 1 Laboratório de Investigação Médica 55 (LIM55), Hospital das Clinicas HCFMUSP, Faculdade de Medicina, Universidade de São Paulo, São Paulo 01246903, SP, Brazil; carolmie96@gmail.com (C.M.M.); patriciacandido11@gmail.com (P.C.); ju.alvcam@gmail.com (J.A.C.); vanessarguima@homail.com (V.R.G.); carolinechiovatto@gmail.com (C.C.); vitoriaghz@gmail.com (V.G.); romaosilva.poliana@gmail.com (P.R.); karinasilva.09@outlook.com (K.S.d.S.); arantes_gabriel@hotmail.com (G.A.d.S.); iransilva@gmail.com (I.A.S.); srougi@srougi.com.br (M.S.); katiaramos@usp.br (K.R.L.); niviana@usp.br (N.I.V.); sabrinareis@usp.br (S.T.R.); 2 D’Or Institute for Research and Education (ID’Or), São Paulo 04501000, SP, Brazil; 3 Campus Santo André, Universidade Federal do ABC, Santo André 09210580, SP, Brazil; 4 Laboratory of Renal Physiology, Department of Physiology and Biophysics, Institute of Biomedical Sciences, University of São Paulo, São Paulo 05508000, SP, Brazil; ggoncalves@usp.br; 5 Campus Ipiranga, Centro Universitário São Camilo, São Paulo 04263200, SP, Brazil; 6 Uro-Oncology Group, Urology Department, Institute of Cancer Estate of São Paulo (ICESP), São Paulo 01246000, SP, Brazil; william.nahas@hc.fm.usp.br; 7 Campus Passos, Universidade do Estado de Minas Gerais—UEMG, Passos 37900106, MG, Brazil

**Keywords:** prostate cancer, hypercholesterolemic model, p160, microRNA

## Abstract

MicroRNAs (miRNAs) have gained a prominent role as biomarkers in prostate cancer (PCa). Our study aimed to evaluate the potential suppressive effect of miR-137 in a model of advanced PCa with and without diet-induced hypercholesterolemia. In vitro, PC-3 cells were treated with 50 pmol of mimic miR-137 for 24 h, and gene and protein expression levels of SRC-1, SRC-2, SRC-3, and AR were evaluated by qPCR and immunofluorescence. We also assessed migration rate, invasion, colony-forming ability, and flow cytometry assays (apoptosis and cell cycle) after 24 h of miRNA treatment. For in vivo experiments, 16 male NOD/SCID mice were used to evaluate the effect of restoring miR-137 expression together with cholesterol. The animals were fed a standard (SD) or hypercholesterolemic (HCOL) diet for 21 days. After this, we xenografted PC-3 LUC-MC6 cells into their subcutaneous tissue. Tumor volume and bioluminescence intensity were measured weekly. After the tumors reached 50 mm3, we started intratumor treatments with a miR-137 mimic, at a dose of 6 μg weekly for four weeks. Ultimately, the animals were killed, and the xenografts were resected and analyzed for gene and protein expression. The animals’ serum was collected to evaluate the lipid profile. The in vitro results showed that miR-137 could inhibit the transcription and translation of the p160 family, SRC-1, SRC-2, and SRC-3, and indirectly reduce the expression of AR. After these analyses, it was determined that increased miR-137 inhibits cell migration and invasion and impacts reduced proliferation and increased apoptosis rates. The in vivo results demonstrated that tumor growth was arrested after the intratumoral restoration of miR-137, and proliferation levels were reduced in the SD and HCOL groups. Interestingly, the tumor growth retention response was more significant in the HCOL group. We conclude that miR-137 is a potential therapeutic miRNA that, in association with androgen precursors, can restore and reinstate the AR-mediated axis of transcription and transactivation of androgenic pathway homeostasis. Further studies involving the miR-137/coregulator/AR/cholesterol axis should be conducted to evaluate this miR in a clinical context.

## 1. Introduction

MicroRNAs (miRNAs) have emerged as an area of great value in biomedical studies, particularly for their influence on gene regulation [1,2] and their association with the control of several biological processes. The aberrant expression of these molecules is related to many diseases, including cancer [3]. For example, epigenetic silencing of tumor suppressor miRNAs is a crucial factor for tumor progression mechanisms of several neoplasms [4], including prostate cancer (PCa) [5]. This downregulation has been associated with increased cell proliferation and invasion, consistent with metastatic phenotypes [6].

Recent theories support the hypothesis that the loss of miR-137, whose promoter region is frequently methylated in PCa, may positively regulate the transcription of androgen-responsive genes, contributing significantly to the clinical progression of the disease [5,7]. These authors also demonstrated that miR-137 has several mRNA targets, including *androgen receptor* (AR) coregulators, such as the p160 family, known to be composed of three *steroid receptor coactivators* (SRC), SRC-1, SRC-2, and SRC-3. Moreover, these genes have been associated with the ability to directly transactivate AR via intrinsic histone acetyltransferase (HAT) activity and have diverse roles in cellular metabolism [8].

Furthermore, bone metastases from castration-resistant prostate cancer (CRPC) with high AR activity exhibit increased metabolic activity compared to those with low androgen activity. In a previous study, we demonstrated that PC-3 cells from bone metastases with the primary site in the prostate, after cholesterol supplementation, exhibited increased expression of cellular metabolism-related genes and proteins [9,10].

Additionally, PCa cells display elevated cholesterol concentrations in their membrane and cytoplasm [11], which have been linked to poor clinical outcomes [12]. Indeed, evidence suggests that the phenomenon involving resistance to first-line therapies for metastatic PCa may be linked to several molecular networks involving AR, AR co-regulatory molecules, and lipid metabolism [13,14,15,16,17].

Previously, our group demonstrated that increased cholesterol levels contribute to p160 coactivator overexpression and indirectly upregulate AR expression, favoring tumor progression [10]. Since miR-137 silencing in advanced PCa may promote overexpression of coactivators and hyperactivation of androgenic signaling, we aimed to demonstrate whether, in the occurrence of androgen axis reactivation in the CRPC phenotype by cholesterol supplementation, miR-137 induction could suppress AR through its coactivators in PC-3 cells.

## 2. Results

### 2.1. Transfection Efficiency with miR-137 Mimic by qPCR

**Figure 1:**
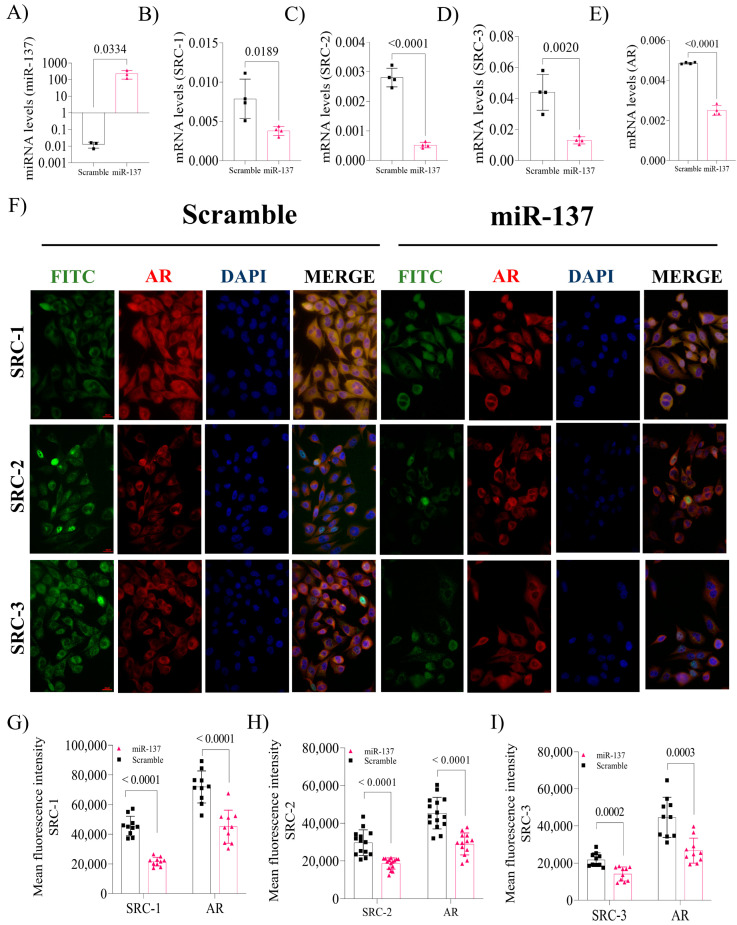
Effect of overexpression of miR-137 in the PC-3 cell line. (**A**) Effectiveness of transfection of miR-137. (**B**) mRNA levels of SRC-1 post-transfection. (**C**) mRNA levels of SRC-2 post-transfection. (**D**) mRNA levels of SRC-3 post-transfection. (**E**) mRNA levels of AR post-transfection. (**F**) Graphical representation of immunofluorescence post-transfection with miR-137 (Magnification 400×). (**G**) Protein levels of SRC-1 and AR. (**H**) Protein levels of SRC-2 and AR. (**I**) Protein levels of SRC-3 and AR. Student’s *t*-test was used for all analyses. The *p*-values obtained from the statistical analyses are shown above the bars in each panel. The error bars correspond to the standard deviation of the samples.

After transfection with the miR-137 mimic, we observed that its expression was 1000-fold higher than in control cells (*p* = 0.033, Figure 1A). After transfection, this miRNA was found to regulate the expression of its target genes negatively, for SRC-1 (*p* = 0.018, Figure 1B), SRC-2 (*p* < 0.0001, Figure 1C), and SRC-3 (*p* = 0.002, Figure 1D). Indirectly, miR-137 negatively influences AR gene expression (*p* = 0.0002, Figure 1E). We also assessed the impact of miR-137 overexpression at the protein level in the PC-3 cell line (Figure 1F). Similarly, SRC-1 (*p* < 0.0001, Figure 1G), SRC-2 (*p* < 0.0001, Figure 1H), and SRC-3 (*p* = 0002, Figure 1I) protein expression was significantly reduced. Simultaneously, AR protein levels were also ascertained and found to be colocalized with p160 family proteins. In this cell, the upregulated miR-137 expression was associated with reduced AR-coactivator levels and, consequently, with attenuated AR protein expression (*p* < 0.0001, Figure 1G–I).

### 2.2. Exogenous Increase of miR-137 in the PC-3 Cells Negatively Regulates Cell Migration and Invasion and Decreases Colony Formation by Altering the Cell Cycle and Inducing Apoptosis

**Figure 2:**
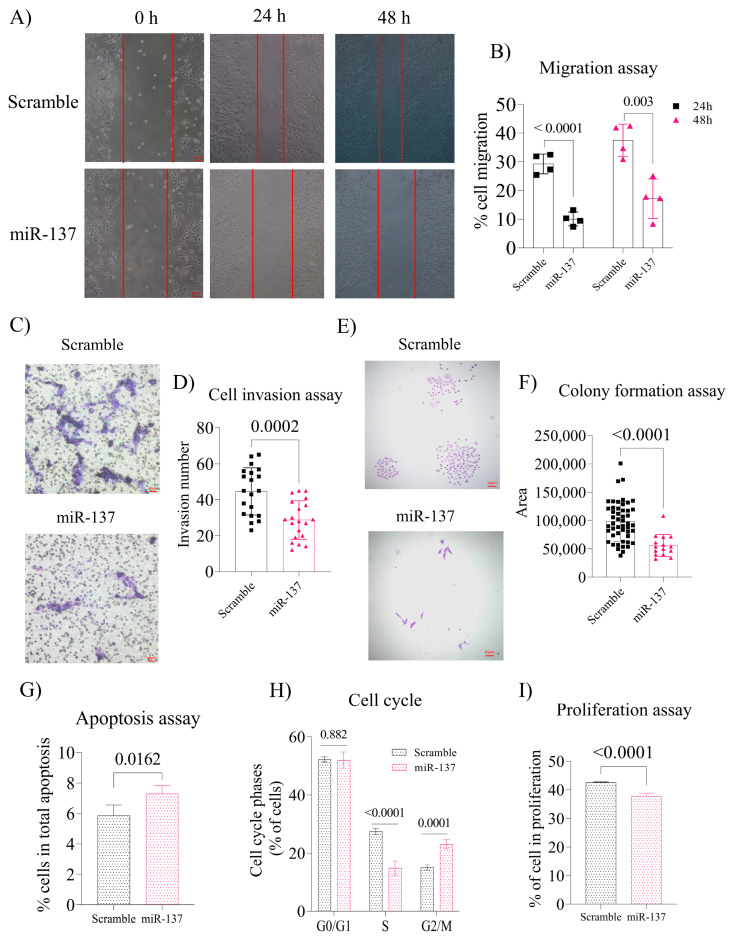
Effect of miR-137 on migration, invasion, colony formation, and flow cytometry assays. (**A**) Representation of the cell migration assay at 0, 24, and 48 h (Magnification 200×). (**B**) The 24 h and 48 h cell migration rates were calculated from time 0 h. The graph represents individual analyses over their respective controls between the 24 h and 48 h times. (**C**) Representation of miR-137 and Scramble cell invasion assay (Magnification 200×). (**D**) Statistical difference between the number of cells in the invasion. (**E**) Representation of the colony formation assay. (**F**) Graphical analysis of the ratio of colony areas posts miR-137 treatment (Magnification 200×). (**G**) Index of apoptosis post-cell transfection. (**H**) Cell cycle assay, G0/G1, S, and G2/M phases. (**I**) Percentage of proliferating cells calculated from the cell cycle’s S and G2 / M phases. Student’s *t*-test was used for all analyses. The *p*-values obtained from the statistical analyses are shown above the bars in each panel. The error bars correspond to the standard deviation of the samples.

Overexpression of miR-137 negatively impacted cell migration potential after 24 and 48 h (*p* < 0.0001 and *p* = 0.003, respectively, Figure 2A,B). The same result was observed in cell invasion ability (*p* = 0.0002, Figure 2C,D) and colony formation ability (*p* < 0.0001, Figure 2E,F). Additionally, it was determined that upregulated miR-137 expression triggers increased apoptosis (*p* = 0.016, Figure 2G) and the number of cells arrested in the G0/G1 phase (*p* = 0.882, Figure 2H), although this result was not statistically significant. However, increased levels of this miR decreased the number of S phase cells (*p* < 0.0001, Figure 2H). The percentage of cells in the G2/M and S phases of the cell cycle indicated that the cell proliferation rate of miR-137-treated cells was significantly reduced compared to control cells (*p* < 0.0001, Figure 2I).

### 2.3. Induction of miR-137 Expression Attenuates Serum Cholesterol Levels and Favors an Increase in HDL, Reducing Prostate Xenograft Tumor Growth

**Figure 3:**
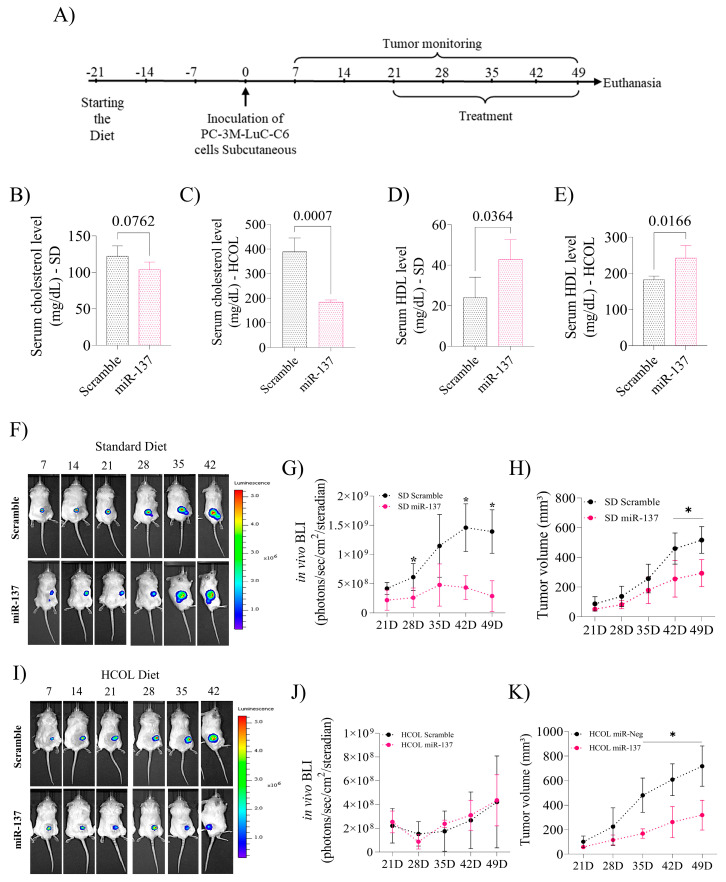
The effect of intratumoral restoration of miR-137 and diet-induced hypercholesterolemia on tumor volume and serum profile of animals. (**A**) Experimental plan. Twenty-one days before xenograft, male NOD / SCID mice were randomly assigned to a standard diet (SD, N = 8) or a hypercholesterolemic diet (HCOL, N = 8). On day 0, cells were injected subcutaneously into the dorsum of the animals. Tumor volume and bioluminescence were measured weekly, and treatment occurred after the tumor volume reached ≥ 50 mm^3^. Subsequently, animals were separated into the following four groups for treatment: SD (Scramble N = 4 and miR-137 N = 4) and HCOL (Scramble N = 4 and miR-137 N = 4). (**B**) Serum cholesterol profile, SD group. (**C**) Serum cholesterol profile, HCOL group. (**D**) HDL levels, SD group. (**E**) HDL levels, HCOL group. (**F**) Bioluminescence measurement by IVIS equipment in the SD group (Scramble vs. miR-137). (**G**) Difference between bioluminescence intensity of Scramble and miR-137 subgroups (SD group). (**H**) Difference between tumor volume of Scramble and miR-137 subgroups, as measured by a digital caliper (SD group). (**I**) Measurement of bioluminescence using IVIS equipment in the HCOL group (Scramble vs. miR-137). (**J**) Difference between bioluminescence intensity of Scramble and miR-137 subgroups (HCOL group). (**K**) Difference between tumor volume of Scramble and miR-137 subgroups, as measured by a digital caliper (HCOL group). Student’s *t*-test was used for all analyses. The *p*-values obtained from the statistical analyses are shown above the bars in each panel. The error bars correspond to the standard deviation of the samples. * = *p*-value ≤ 0.05.

The mice were killed at the end of the experiments, and serum was collected for biochemical analyses (Figure 3A). Interestingly, when subdivided between the miR-137 and Scramble groups, we observed that SD and HCOL animals treated with miR-137 tended to present reduced serum cholesterol levels (*p* = 0.076, Figure 3B and *p* = 0.0007, Figure 3C, respectively). There was an increase in serum HDL levels in the SD and the HCOL subgroups treated with miR-137 (*p* = 0.036 and *p* = 0.016, Figure 3D,E, respectively).

Bioluminescence analysis revealed that the SD group that received miR-137 as a form of treatment demonstrated a decrease in cellular activity captured by the IVIS instrument (*p* < 0.05, Figure 3F,G). Furthermore, tumor growth velocity, analyzed by digital pachymeter, was significantly reduced in these same animals (*p* < 0.05, Figure 3H). The HCOL group showed no difference in bioluminescence captured by the IVIS device (*p* < 0.05, Figure 3I,J). However, after tumor measurement, animals receiving miR-137 had smaller tumor volumes (*p* < 0.05, Figure 3K). The body weight of the groups and subgroups showed no differences throughout the experiment.

### 2.4. Intratumoral Reinstatement of the miR-137 Leads to Reduced Expression of p160 Family Proteins and Negatively Impacts AR Expression in Advanced PCa Xenografts, Leading to Reduced Tissue Proliferation

**Figure 4:**
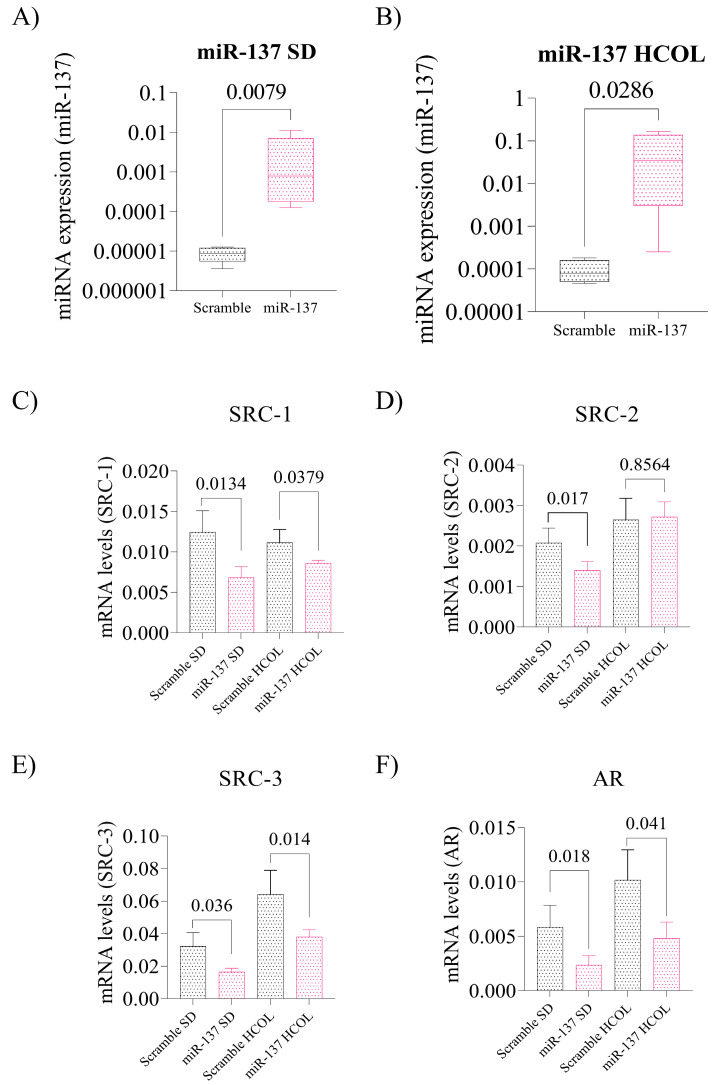
Expression levels of miR-137 and its target mRNAs from the post-treatment tumor xenografts. (**A**) Expression levels of miR-137 in the SD group; Mann–Whitney test. (**B**) Expression levels of miR-137 in the HCOL group; Mann–Whitney test. (**C**) Expression levels of SRC-1 mRNA in the SD and HCOL groups post miR-137 treatment; Student’s *t*-test. (**D**) SRC-2 mRNA expression levels in the SD and HCOL groups post miR-137 treatment; Student’s *t*-test. (**E**) SRC-3 mRNA expression levels in the SD and HCOL groups post miR-137 treatment; Student’s *t*-test. (**F**) AR mRNA expression levels in the SD and HCOL groups post miR-137 treatment; Student’s *t*-test. Statistical analyses for p160 and AR genes were performed individually, with their control and miR within their data group, SD (Scramble x miR—bars on the left) and HCOL (Scramble x miR—bars on the right). The *p*-values obtained from the statistical analyses are shown above the bars in each panel. The error bars correspond to the standard deviation of the samples.

The expression of miR-137 was examined by qPCR in the tumors from the animals in the SD and HCOL groups. We observed a significant upregulation of miR-137 in the experimental tumors compared to the tumors from the Scramble group (*p* = 0.007, Figure 4A and *p* = 0.028, Figure 4B, respectively). When analyzing the expression of p160 family genes and AR in the SD group, there was a significant reduction for all genes when compared to the SD Scrambles (SRC-1: *p* = 0.013, Figure 4C; SRC-2: *p* = 0.017, Figure 4D; SRC-3: *p* = 0.036, Figure 4E; and AR: *p* = 0.018, Figure 4F). In the HCOL group, SRC-1 (*p* = 0.037, Figure 4C), SRC-3 (*p* = 0.014, Figure 4E), and AR (*p* = 0.041, Figure 4F) gene expression was decreased after miR-137 treatment.

**Figure 5:**
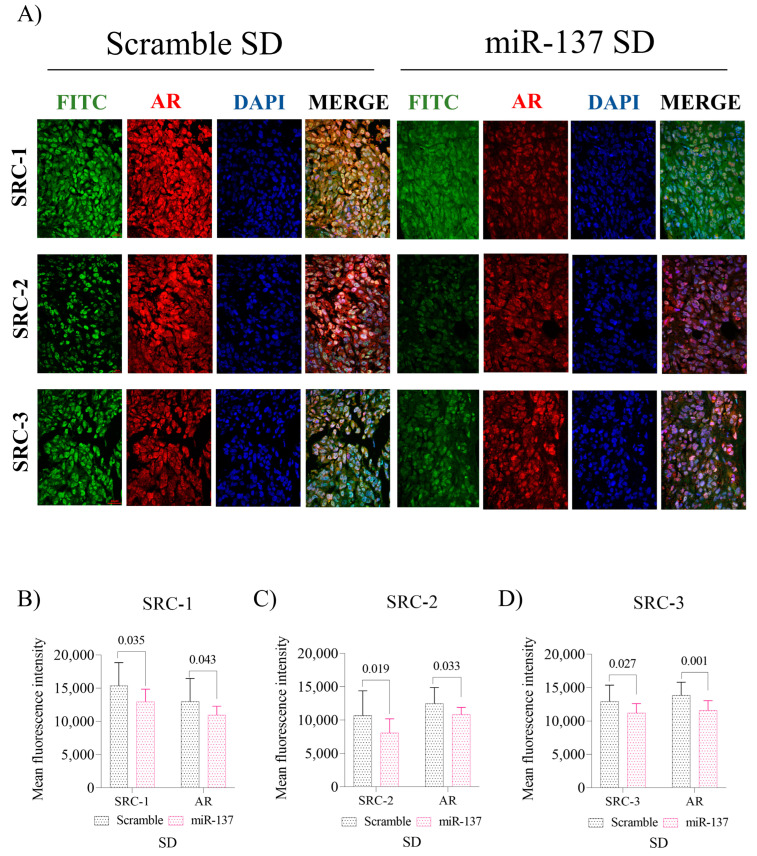
In vivo effect of miR-137 and cholesterol restoration on p160 and AR protein expression, SD group. (**A**) Representation of IF in tissues immunolabeled with anti-SRC-1, SRC-2, SRC-3, and AR treated with miR-137 and their respective control (Magnification 400×). (**B**) Fluorescence intensity levels of SRC-1 and AR. (**C**) Fluorescence intensity levels of SRC-2 and AR. (**D**) Fluorescence intensity levels of SRC-3 and AR. Student’s *t*-test was used in all analyses. The *p*-values obtained from the statistical analyses are shown above the bars in each panel. The error bars correspond to the standard deviation of the samples.

Concerning protein levels in the SD group, we found that the p160 family proteins (Figure 5A), SRC-1 (*p* = 0.035, Figure 5B), SRC-2 (*p* = 0.019, Figure 5C), and SRC-3 (*p* = 0.027, Figure 5D) were significantly reduced. As shown in Figure 5B–D, increasing miR-137 target expression reduces AR protein expression indirectly, as evidenced by double labeling with SRC-1, SRC-2, and SRC-3 antibodies (*p* < 0.05).

**Figure 6:**
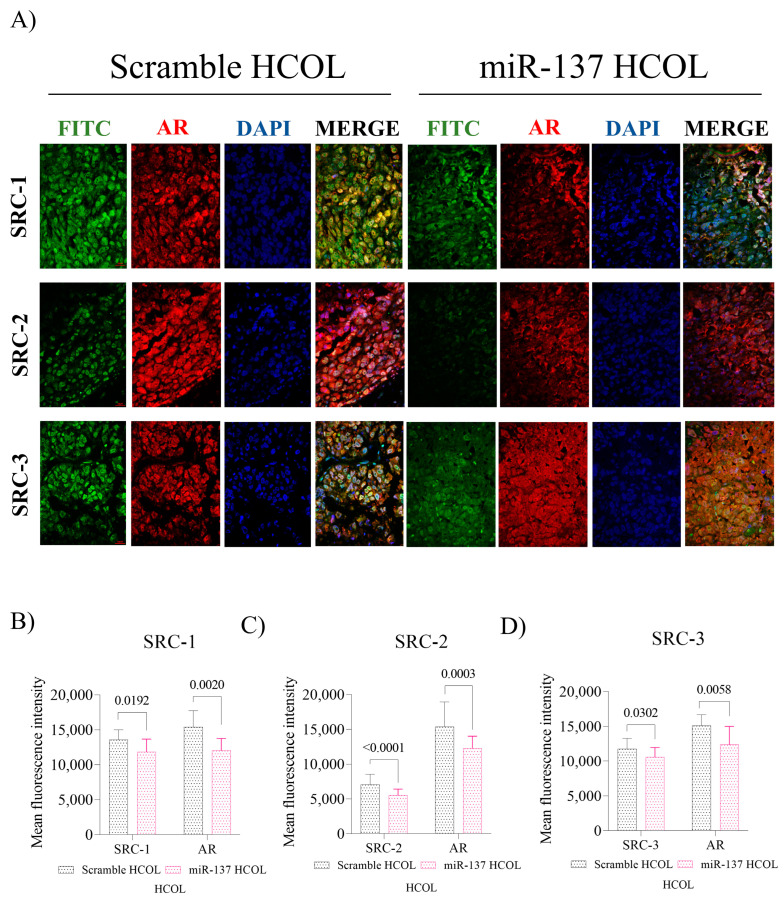
In vivo effect of miR-137 and cholesterol restoration on p160 and AR protein expression, HCOL group. (**A**) Representation of IF in tissues immunolabeled with anti-SRC-1, SRC-2, SRC-3, and AR treated with miR-137 and their respective control (Magnification 400×). (**B**) Fluorescence intensity levels of SRC-1 and AR. (**C**) Fluorescence intensity levels of SRC-2 and RA. (**D**) Fluorescence intensity levels of SRC-3 and RA; Mann–Whitney test. Student’s *t*-test was used in all analyses except AR/SRC-3. The *p*-values obtained from the statistical analyses are shown above the bars in each panel. The error bars correspond to the standard deviation of the samples.

**Figure 7:**
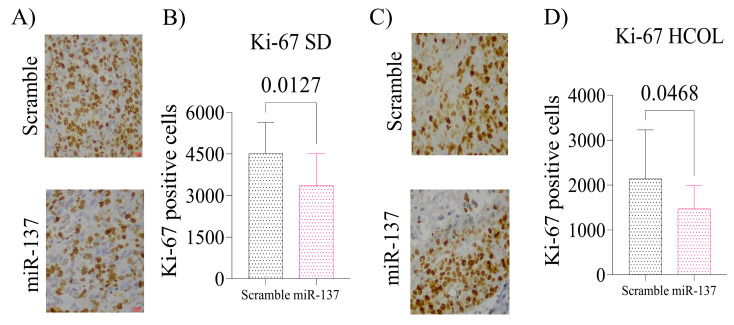
Tissue proliferation index by Ki-67. Cell proliferation levels were measured by Ki-67 immunohistochemistry reaction (Clone Mib-1). (**A**) Representative images of the SD group after being treated with miR-137 and control (Magnification 200×). (**B**) Statistical analysis of tissue proliferation in the SD group. (**C**) Representative images of the HCOL group after being treated with miR-137 and control (Magnification 200×). (**D**) Statistical analysis of tissue proliferation in the HCOL group. Student’s *t*-test was used for all analyses. The *p*-values obtained from the statistical analyses are shown above the bars in each panel. The error bars correspond to the standard deviation of the samples.

The HCOL group also displayed reduced SRC-1 (*p* = 0.019, Figure 6B), SRC-2 (*p* < 0.0001, Figure 6C), SRC-3 (*p* = 0.032, Figure 6D), and AR (*p* < 0.05, Figure 6B–D) protein expression. Additionally, after decreasing the expression of the p160 family and AR, tumor cell proliferation in xenografts of the SD miR-137 group was reduced compared to Scramble (*p* = 0.012, Figure 7A,B). The same result was observed for the HCOL miR-137 group (*p* = 0.046, Figure 7C,D).

## 3. Discussion

In recent decades, there has been considerable interest in the potential clinical value of miRNAs in PCa [18], especially by our research group [19,20,21,22,23,24,25,26,27,28,29,30,31,32]. Eedunuri et al. (2015) [7] showed that miR-137 negatively regulates the three genes comprising the p160 family. Data published in the same year reported that miR-137 is partially methylated in PCa, with more aggressive phenotypes containing heavy methylation where this miRNA is encoded [5].

SRC-1, SRC-2, and SRC-3 genes are known to mediate transcription functions of nuclear receptors and other transcription factors and play crucial roles in hormonal, proliferative, metastatic, and therapeutic resistance signaling. Previously, in tumor xenografts (PC-3 cells) from an animal model of CRPC with diet-induced hypercholesterolemia, we demonstrated that cholesterol could trigger the translocation of these proteins, upregulate their expression and increase AR expression, which was associated with tumor progression [10]. In this study, we demonstrated that miR-137, even in the presence of cholesterol, could control tumor growth rate and decrease cell proliferation by modulating its targets.

In the in vitro experiments, using the PC-3 cells, we demonstrated that miR-137 could attenuate the transcription and translation of its target. Restoring miR-137 in the PC-3 cell line decreased cell migration and invasion, negatively impacting the S phase of the cell cycle, leading to reduced proliferation and increased apoptosis rates, which corroborates data available in the literature [5,7]. Indirectly, miR-137 also downregulated AR levels in the same cell line. It is plausible that this event is associated with reduced coactivator expression.

We used the diet-induced hypercholesterolemia model utilized previously [10] to ascertain the combined effect of miR-137 and cholesterol. Intratumoral restoration of miR-137 decreased the speed of tumor growth in both SD and HCOL groups. Interestingly, increased miR-137 reduced serum cholesterol concentrations and increased serum HDL. Although data demonstrate the role of various miRNAs in cholesterogenic and lipogenic pathways [33], we found no data in the literature that could explain the role of miR-137 in this context. Since the xenografts are vascularized, significant portions of this miRNA may move into the vessels, triggering lipid metabolism and cholesterogenic pathway regulation. However, further studies should be performed better to understand the role of miR-137 in these pathways.

Interestingly, we observed that in the HCOL group, miR-137 reduced growth velocity more effectively than in the SD group between days 35, 42, and 49. We hypothesize that the cholesterol may have enhanced the restoration response of this miRNA. Corroborating our findings, Nilsson et al. (2015) reported that miR-137 responds to androgen induction and is epigenetically silenced in prostate tumors, hemimethylated in androgen-responsive cells (e.g., LNCaP), and methylated in non-responsive cells (e.g., PC-3) [5]. Previously, our group demonstrated that cholesterol positively regulated SRC-1, SRC-2, and SRC-3 coactivators [10]. Here, we show that miR-137 treatment in the same model (i.e., CRPC with diet-induced hypercholesterolemia) was able to negatively impact the expression of p160 coactivators and delay tumor progression. Thus, miR-137 regulatory activity is potentialized by the presence of androgens directly or by its precursors (e.g., cholesterol). However, the absence or decrease of this miRNA can lead to AR hyperactivation and signaling.

In summary, the present study demonstrated that miR-137 restoration is an attractive therapeutic alternative for PCa, especially the CRPC phenotype. Our results show that androgen signaling triggered by dietary cholesterol can help restore the homeostasis of the miR-137/coactivators/AR axis. Furthermore, this study provides insights into novel molecularly targeted therapeutic ways of utilizing miRNAs to target specific diseases.

## 4. Materials and Methods

### 4.1. TargetScan, miRDB, and miRmap Databases

To identify possible binding regions between miR-137 and its putative target genes, SRC-1, SRC-2, and SRC-3, we utilized the following three online databases: TargetScan [34], miRDB [35], and miRmap [36] (Supplementary Tables S1, S2, S3, and S4, respectively; Supplementary Figure S1). These databases provided reliable analyses of potential miRNA–mRNA interactions. Using the default parameters, the tools were employed, and binding regions with high confidence scores were predicted. Subsequently, the data obtained were validated by a previously published study [7] (Supplementary Figure S2), which demonstrated the interaction between miR-137 and the three genes comprising the p160 coactivator family.

### 4.2. Cell Transfection

PC-3M-luc-C6 (PC-3) cells were seeded in 24-well culture plates at 8 × 10^5^ cells/well and grown in MEM medium (Invitrogen, Grand Island, NY, USA) using standard culture methods for miRNA transfection. The cell line was authenticated by Short Tandem Repeat (STR) (Supplementary Figure S3). The transfection into the PC-3 cell line was carried out in Opti-MEM I using Lipofectamine RNAiMAX (Thermo Fisher Scientific, Waltham, MA, USA) according to the manufacturer’s protocol. Synthetic miR-137 (assay ID MC10513-Ambion) and its respective Scramble control (ctrl miR; catalog number AM17110) were used at a final concentration of 50 pmol. All PC-3 cell line assays were performed in triplicate.

### 4.3. RNA Extraction and Quantitative Real-Time Polymerase Chain Reaction

According to the manufacturer’s instructions, cellular RNAs were extracted using the mirVana kit (Ambion, Austin, TX, USA). Total RNA was used to synthesize complementary DNA (cDNA) using a cDNA High-Capacity Reverse Transcription Kit (Applied Biosystems, Foster City, CA, USA). The miRNA cDNA was generated using a TaqMan miRNA Reverse Transcription Kit (Applied Biosystems). The target gene sequences were amplified in a 10 µL reaction mixture containing 5 µL of TaqMan Universal PCR Master Mix and 0.5 µL of TaqMan Gene Expression (Supplementary Table S5). GAPDH was the endogenous control in the gene expression analyses, and RNU48 was the endogenous control in the miRNA analyses. Data were analyzed using Data Assist Software (version v3.01) (Applied Biosystems). All qPCR reactions were performed in duplicate.

### 4.4. Apoptosis and Cell Cycle Analyses by Flow Cytometry

Following transfection with miR-137, flow cytometry experiments were performed on a Muse Cell Analyzer (Merck Millipore, Burlington, MA, USA). After 24 h, the cells were labeled with Muse Annexin V & Cell Death (MCH100105) and Muse Cellular Cycle (MCH100105) kits according to the manufacturer’s recommendations.

### 4.5. Cell Migration Assay

Following transfection with miR-137, PC-3 cells were transferred to 24-well plates with 5 × 10^4^ cells/well. After reaching confluence, a wound was made (represented by the risk). Then, the cells were washed, and MEM medium without FBS was added to each well. Each well was photographed at 0, 24, and 48 h. The images were analyzed using NIS Elements D 3.1 software (Nikon Eclipse E200, Tokyo, Japan). Percent migration was calculated based on the risk area at 0, 24, and 48 h.

### 4.6. Colony Formation Assay

Cells were transferred at low density (6 × 10^2^ cells/well) to 12-well plates and transfected with miR-137 as previously described. Cells were incubated at 37 °C and 5% CO_2_ for 7 d. Cells were washed with PBS for staining, then 1 mL of violet crystal (0.1%) was added and incubated for 30 min. The plates were washed, and then a colony count was performed. The plates were photographed, and the images were analyzed using ImageJ software (version v.1.53k).

### 4.7. Invasion Assays

Using the PC-3 cell line, the invasion was assessed by counting the number of cells that invaded the lower portion of Transwell chambers (Greiner Bio-one, Americana, SP, Brazil) with a pore size of 8 μm containing 50 mL of Matrigel diluted in serum-free culture medium (1:2), after transfection with miR-137 precursor. In this step, 1 × 10^4^ cells/mL in serum-free culture medium were seeded into the Matrigel, and 750 µL of culture medium containing FBS (10%) was added to the lower chamber. The cells were maintained in a CO_2_ incubator for 24–48 h at 37 °C. The cells were fixed with formaldehyde in PBS (4%), stained with crystal violet solution (0.2%) in methanol, and counted using an optical microscope at 200× magnification.

### 4.8. Immunofluorescence In Vitro

For the immunofluorescence (IF) assay, the cells were cultured on glass coverslips and transfected with miR-137 and its respective control (Scramble) to evaluate AR-co-activating protein expression. The cells were fixed with 4% paraformaldehyde in 1× PBS for 10 min, permeabilized with 0.1% Triton X-100 in 1× PBS for five more minutes and blocked with 2.5% Normal Horse Serum for 90 min (Vector Laboratories, Burlingame, CA, USA). Coverslips were incubated with the primary antibodies of the proteins SRC-1, SRC-2, SRC-3, and AR (Supplementary Table S6), and diluted 1:100 in Normal Horse Serum (2.5%) for 12 h, followed by the secondary antibody diluted for 1 h (VectaFluor Duet Immunofluorescence Double Labeling Kit, DyLight 488 Anti-Rabbit (green), DyLight 594 Anti-Mouse (red)), according to methods described in a previous study [10]. Coverslips were washed three times in 1× PBS after each antibody incubation. Where indicated, coverslips were counterstained with DAPI (Cell Signaling, Danvers, MA, USA), washed three times with 1× PBS, and mounted using ProLong Diamond (Invitrogen). Cells were photographed using a fluorescence microscope (Eclipse 80i, Nikon, Tokyo, Japan) attached to a photographic camera. ImageJ software (version 1.53k) quantified the fluorescence signal and reported it as mean fluorescence intensity.

### 4.9. In Vivo Study

The in vivo study was approved by the institution’s Ethics and Animal Research Committee (Protocol # 1360/2019). The Bioterio Central—FMUSP (São Paulo, SP, Brazil) provided 16 four to five-week-old male NOD/SCID mice. All animal manipulations were performed under sterile conditions. The animals were divided into two groups, hypercholesterolemic and control. The hypercholesterolemic group (HCOL; N = 8) was fed a high-calorie diet (2% cholesterol—PragSoluções, Jaú, SP, Brazil), and the control group was fed a standard diet (SD; N = 8) (AIN-76—PragSoluções). Twenty-one days after starting the respective diets, mice were placed under isoflurane anesthesia, and 1.5 × 10^6^ PC-3 cells (final volume of 50 µL of medium) were injected into the subcutaneous tissue, as described previously [10]. When the mean tumor volumes reached 50 mm^3^, HCOL and SD mice were randomly subdivided into Scramble (N = 4) and miR-137 (N = 4). Then, 6 µg of miR-137 mimic and Scramble were complexed with in vivo-jetPEI^®^ (Polyplus-Transfection, New York, NY, USA) in 30 µL of a 5% glucose solution following the manufacturer’s recommendations. The complex was injected intratumorally at 7-day intervals for a total of four times. The mice continued to consume their respective diets until being killed (i.e., 30 days after cell injection). Tumors were palpable, and the length (L) and width (W) were measured with a digital caliper. The tumor volume (V) was calculated every 2 d using the following formula: V = [W × L × (W + L)/2)] × 0.52 [10].

Immediately after the PC-3 xenografts, D-luciferin (Promega, Madson, Oceanside, CA, USA) (5 mg/kg) was injected into the peritoneum to assess the inoculation efficiency into the animals’ subcutaneous tissue using an IVIS Spectrum imaging system (Perkin Elmer, Santa Clara, CA, USA). Typically, tumors were palpable between 7 and 14 d after the PC-3 cell inoculation.

### 4.10. Lipid Profile

Both groups’ serum cholesterol and HDL levels were measured using Labtest kits (Labtest Diagnostica, Lagoa Santa, MG, Brazil). The same kit was used to measure intratumoral cholesterol levels in tissue homogenates. The absorbance of the samples was recorded in a SpectraMax 340PC384 microplate reader (Molecular Devices, San Jose, CA, USA) following the manufacturer’s recommendations.

### 4.11. Immunohistochemical Assay

The paraffinized samples were subjected to a heat antigen retrieval process using citrate buffer (1 mM, pH 6.0). Subsequently, the slides were incubated overnight at 4 °C with an anti-Ki-67 antibody (Clone mib-1). The LSAB system was used for immunostaining (Dako Cytomation, Santa Clara, CA, USA). The color was developed by reacting with a 3,3′diaminobenzidine solution of the chromogenic substrate, followed by counterstaining with Harris hematoxylin. At the end of the reactions, the slides were observed and photographed under a light microscope, and quantification was performed using ImageJ software (version v.1.53k) and reported as mean fluorescence intensity.

### 4.12. Immunofluorescence In Vivo

Paraffin-fixed sections of the animals’ xenografts were cut into 4 μm slices, then deparaffinized and blocked with 2.5% Normal Horse Serum (Vector Laboratories) for 1 h at room temperature before being incubated overnight at 4 °C with the anti-SRC-1, anti-SRC-2, anti-SRC-3, and anti-AR antibodies (Supplementary Table S3). Then, the xenograft sections were incubated with a VectaFluor Duet Immunofluorescence Double Labeling Kit, DyLight 488 Anti-Rabbit (green) and DyLight 594 Anti-Mouse (red) for 1 h at room temperature in the dark and mounted with Fluoroshield (Sigma Aldrich, Saint Louis, MI, USA). Protein staining was analyzed using a Zeiss LSM 510 confocal microscope with a 40× objective and laser excitation at 488 or 594 nm. At least five fields per slide were analyzed. ImageJ software (version v.1.53k) was used to quantify the fluorescence signal as intensity (arbitrary units (AU)).

### 4.13. Statistical Analysis

Statistical analyses were performed using GraphPad Prism 9.0 software. All in vitro experiments were performed in biological triplicates. The Shapiro-Wilk test was used to assess the normality of the data, and Student’s *t*-tests and Mann–Whitney tests were used to compare the in vitro and in vivo data. The level of statistical significance was set to 5% (*p* ≤ 0.05).

## 5. Conclusions

We conclude that miR-137 is a potent transcriptional and translational inhibitor of the p160 family and can repress critical regulatory tumor progression pathways in the CRPC phenotype. Diet-induced hypercholesterolemia in an environment with little or no miR-137 expression significantly impacts tumor progression. In conjunction with restoring miR-137 expression, hypercholesterolemia may contribute to miR-137/coactivators p160/AR axis homeostasis. From a therapeutic perspective, modulating the expression of this miRNA may contribute considerably to the future of precision medicine, especially urologic oncology.

## Data Availability

The datasets used and/or analyzed during the current study are available from the corresponding author upon reasonable request.

## Supplementary Materials

The following supporting information can be downloaded at https://www.mdpi.com/article/10.3390/ijms24119633/s1.
