# Supplementary material for: Intratumoral Restoration of miR-137 Plus Cholesterol Favors Homeostasis of the miR-137/Coactivator p160/AR Axis and Negatively Modulates Tumor Progression in Advanced Prostate Cancer
Source: Int J Mol Sci. 2023 Jun 1;24(11):9633. doi: 10.3390/ijms24119633 (PMC10253647; doi:10.3390/ijms24119633)
Supplement: Supplementary file 1 [file ijms-24-09633-s001.zip › ijms-2367898-supplementary.pdf]

Table S1. Binding regions between miR-137 and its putative target genes, SRC-1, SRC-2, and SRC-3 (TargetScan).

Name	Total	microRNAs
SRC-1; SRC-2; SRC-3	208	hsa-miR-4743-3p hsa-miR-557 hsa-miR-3690 hsa-miR-6891-5p hsa-miR-377-5p hsa-miR-562 hsa-miR-3681-3p hsa-miR-6780b-5p hsa-miR-23a-3p hsa-miR-489-3p hsa-miR-2110 hsa-miR-4659a-3p hsa-miR-8485 hsa-miR-3653-3p hsa-miR-6511a-5p hsa-miR-4668-5p hsa-miR-205-5p hsa-miR-4477a hsa-miR-1277-5p hsa-miR-4735-3p hsa-miR-507 hsa-miR-6883-5p hsa-miR-4731-5p hsa-let-7g-3p hsa-miR-1301-5p hsa-miR-551b-5p hsa-miR-603 hsa-miR-4505 "Poorly conserved sites hsa-miR-513b-5p hsa-miR-875-3p hsa-miR-4268 hsa-miR-338-5p hsa-miR-200c-3p hsa-miR-4282 hsa-miR-4652-3p hsa-miR-1285-3p hsa-miR-548az-5p hsa-miR-4725-3p hsa-miR-5692c hsa-miR-548t-3p hsa-miR-149-3p hsa-miR-1236-3p hsa-miR-4680-3p hsa-miR-6128 hsa-miR-590-3p hsa-miR-5589-5p hsa-miR-548ae-3p hsa-miR-4728-5p hsa-miR-23c hsa-miR-708-3p hsa-miR-33a-5p hsa-miR-4699-3p hsa-miR-4509 hsa-miR-23b-3p hsa-miR-5004-3p hsa-miR-429 hsa-miR-548aa hsa-miR-5692b hsa-miR-612 hsa-miR-126-5p hsa-miR-488-5p " hsa-miR-362-3p hsa-miR-3646 hsa-miR-548ah-3p hsa-miR-128-3p hsa-miR-548ap-3p hsa-miR-7856-5p hsa-miR-4446-5p hsa-miR-4517 hsa-miR-3128 hsa-miR-145-3p hsa-miR-329-3p hsa-miR-548t-5p hsa-miR-6785-5p hsa-miR-4330 hsa-miR-8067 hsa-miR-130b-5p hsa-miR-1250-3p hsa-miR-548f-5p hsa-miR-7703 hsa-miR-340-5p hsa-miR-3942-3p hsa-miR-548aq-3p hsa-miR-3679-3p hsa-miR-7850-5p hsa-miR-369-3p hsa-miR-410-3p hsa-miR-548x-3p hsa-miR-520d-5p hsa-miR-4653-3p hsa-miR-1252-3p hsa-miR-6809-3p hsa-miR-27a-3p hsa-miR-3124-3p hsa-miR-33b-5p hsa-miR-365b-5p hsa-miR-548aj-3p hsa-miR-548ah-5p hsa-miR-7-2-3p hsa-miR-3123 hsa-miR-181a-2-3p hsa-miR-513a-5p hsa-miR-3163 hsa-miR-18b-5p hsa-miR-18a-5p hsa-miR-9-5p hsa-miR-216a-3p hsa-miR-7-1-3p hsa-miR-374a-5p hsa-miR-3662 hsa-miR-29b-2-5p hsa-miR-514b-5p hsa-miR-3607-3p hsa-miR-6860 hsa-miR-3613-3p hsa-miR-597-3p hsa-miR-548x-5p hsa-miR-493-5p hsa-miR-3934-5p hsa-miR-655-3p hsa-miR-6508-5p hsa-miR-548m hsa-miR-182-3p hsa-miR-3173-3p hsa-miR-4448 hsa-miR-544a-3p hsa-miR-365a-5p hsa-miR-548n hsa-miR-3191-5p hsa-miR-5688 hsa-miR-4753-3p hsa-miR-5787 hsa-miR-195-3p hsa-miR-548aj-5p hsa-miR-506-5p hsa-miR-524-5p hsa-miR-4659b-3p hsa-miR-200b-3p hsa-miR-29a-5p hsa-miR-3941 hsa-miR-26b-3p hsa-miR-4796-3p hsa-miR-4482-3p hsa-miR-6502-5p hsa-miR-3148 hsa-miR-4762-3p hsa-miR-1207-3p hsa-miR-362-5p hsa-miR-6802-3p hsa-miR-382-3p hsa-miR-4796-5p hsa-miR-5187-3p hsa-miR-143-5p hsa-miR-6875-3p hsa-miR-6750-3p hsa-miR-4795-3p hsa-miR-374b-5p hsa-miR-4427 hsa-miR-545-3p hsa-miR-548j-3p hsa-miR-3913-3p hsa-miR-651-3p hsa-let-7a-2-3p hsa-miR-3187-5p hsa-miR-3121-3p hsa-miR-4271 hsa-miR-4254 hsa-miR-607 hsa-miR-7851-3p hsa-miR-578 hsa-miR-4766-5p hsa-miR-548am-3p hsa-miR-3609 hsa-miR-548aw hsa-miR-1468-3p hsa-miR-4719 hsa-miR-361-3p hsa-miR-5189-5p hsa-let-7c-3p hsa-miR-335-5p hsa-miR-16-2-3p hsa-miR-4766-3p hsa-miR-1245b-3p hsa-miR-1299 hsa-miR-548c-3p hsa-miR-1283 hsa-miR-374c-5p hsa-miR-137 hsa-miR-130a-5p hsa-miR-6888-5p hsa-miR-6507-5p hsa-miR-4775 hsa-miR-495-3p hsa-miR-1910-3p hsa-miR-152-5p hsa-miR-4666a-3p hsa-miR-3920 hsa-miR-5580-3p hsa-miR-655-5p hsa-

		miR-6885-3p hsa-miR-27b-3p hsa-miR-548g-5p hsa-miR-513c-5p hsa-miR-6086 hsa-miR-6124 hsa-miR-3671
SRC-1; SRC-2	164	hsa-miR-522-5p hsa-miR-518d-5p hsa-miR-4674 hsa-miR-448 hsa-miR-3140-5p hsa-miR-103a-2-5p hsa-miR-3977 hsa-miR-518e-5p hsa-miR-513a-3p hsa-miR-223-3p hsa-miR-7113-5p hsa-miR-3908 hsa-miR-186-3p hsa-miR-4257 hsa-miR-6766-3p hsa-miR-5584-5p hsa-miR-3149 hsa-miR-6841-3p hsa-miR-4777-5p hsa-miR-8071 hsa-miR-23a-5p hsa-miR-8055 hsa-miR-3612 hsa-miR-1207-5p hsa-miR-4438 hsa-miR-6880-5p hsa-miR-5003-3p hsa-miR-586 hsa-miR-3692-3p hsa-miR-627-3p hsa-miR-525-5p hsa-miR-4457 hsa-miR-125b-2-3p hsa-miR-3119 hsa-miR-3606-3p hsa-miR-569 hsa-miR-4731-3p hsa-miR-6782-5p hsa-miR-6757-3p hsa-miR-6514-5p hsa-miR-6857-3p hsa-miR-4802-5p hsa-miR-654-3p hsa-miR-508-3p hsa-miR-4677-5p hsa-miR-8089 hsa-miR-6864-5p hsa-miR-6782-3p hsa-miR-4259 hsa-miR-6754-5p hsa-miR-1257 hsa-miR-4667-5p hsa-miR-567 hsa-miR-4728-3p hsa-miR-937-5p hsa-miR-526a hsa-miR-6828-3p hsa-miR-501-5p hsa-miR-5702 hsa-miR-3185 hsa-miR-4270 hsa-miR-519a-5p hsa-miR-7111-5p hsa-miR-3675-3p hsa-miR-4700-5p hsa-miR-339-5p hsa-miR-4782-3p hsa-miR-135a-5p hsa-miR-3922-5p hsa-miR-1587 hsa-miR-764 hsa-miR-518f-5p hsa-miR-626 hsa-miR-144-5p hsa-miR-4434 hsa-miR-6870-5p hsa-miR-944 hsa-miR-219a-5p hsa-miR-3121-5p hsa-miR-6811-3p hsa-miR-520g-5p hsa-miR-3652 hsa-miR-4428 hsa-miR-3179 hsa-miR-539-5p hsa-miR-8084 hsa-miR-8081 hsa-miR-6876-3p hsa-miR-3180-5p hsa-miR-5698 hsa-miR-4477b hsa-miR-222-5p hsa-miR-4768-5p hsa-miR-4777-3p hsa-miR-335-3p hsa-miR-27a-5p hsa-miR-650 hsa-miR-3140-3p hsa-miR-4325 hsa-miR-3976 hsa-miR-8075 hsa-miR-6825-5p hsa-miR-4443 hsa-miR-4430 hsa-miR-3942-5p hsa-miR-4699-5p hsa-miR-3605-5p hsa-miR-548ay-3p hsa-miR-4763-3p hsa-miR-520a-5p hsa-miR-523-5p hsa-miR-6770-5p hsa-miR-4441 hsa-miR-3620-5p hsa-miR-502-5p hsa-miR-1233-3p hsa-miR-6847-5p hsa-miR-6833-3p hsa-miR-498 hsa-miR-5681b hsa-miR-4445-5p hsa-miR-4786-3p hsa-miR-23b-5p hsa-miR-568 hsa-miR-5703 hsa-miR-184 hsa-miR-135b-5p hsa-miR-125a-3p hsa-miR-520c-5p hsa-miR-6868-5p hsa-miR-6842-3p hsa-miR-4742-3p hsa-miR-452-3p hsa-miR-548at-3p hsa-miR-4670-3p hsa-miR-4708-5p hsa-miR-4703-5p hsa-miR-659-3p hsa-miR-32-3p hsa-miR-2467-3p hsa-miR-4279 hsa-miR-1827 hsa-miR-3165 hsa-miR-4639-5p hsa-miR-297 hsa-miR-412-3p hsa-miR-377-3p hsa-miR-218-1-3p hsa-miR-4804-5p hsa-miR-4516 hsa-miR-409-3p hsa-miR-513c-3p hsa-miR-519b-5p hsa-miR-33a-3p hsa-miR-548g-3p hsa-miR-4461 hsa-miR-519c-5p hsa-miR-4801 hsa-miR-873-5p.1 hsa-miR-548as-3p hsa-miR-6754-3p hsa-miR-4723-5p hsa-miR-1225-3p hsa-miR-4643
SRC-1; SRC-3	162	hsa-miR-140-5p hsa-miR-637 hsa-miR-3622b-3p hsa-miR-6887-5p hsa-miR-515-5p hsa-miR-92b-3p hsa-miR-5089-5p hsa-miR-3622a-3p hsa-miR-5692a hsa-miR-892c-5p hsa-miR-26a-2-3p hsa-miR-34a-3p hsa-miR-4513 hsa-miR-661 hsa-miR-499b-3p hsa-miR-5186 hsa-miR-4799-5p hsa-miR-95-5p hsa-miR-1-3p hsa-miR-6787-3p hsa-miR-32-5p hsa-miR-298 hsa-let-7b-5p hsa-miR-6735-5p hsa-miR-302b-5p hsa-miR-4436b-3p hsa-miR-211-3p hsa-miR-320e hsa-miR-4312 hsa-miR-6731-3p hsa-miR-3912-5p hsa-let-7g-5p hsa-miR-3655 hsa-miR-378a-5p hsa-miR-519e-5p hsa-miR-2355-3p hsa-miR-5697 hsa-miR-4682 hsa-miR-3661 hsa-miR-4632-5p hsa-miR-301a-3p hsa-miR-372-5p hsa-miR-1200 hsa-miR-3199 hsa-miR-608 hsa-miR-4698 hsa-let-7i-5p hsa-miR-1273g-3p hsa-miR-1252-5p hsa-miR-500b-3p hsa-miR-584-3p hsa-miR-26a-1-3p hsa-miR-5696 hsa-miR-6881-3p hsa-miR-6721-5p hsa-miR-7843-5p hsa-miR-6882-3p hsa-miR-548e-5p hsa-miR-548q hsa-miR-6855-3p hsa-miR-7151-5p hsa-miR-6757-5p hsa-miR-7114-5p hsa-miR-642a-3p hsa-let-7f-5p hsa-miR-6842-5p hsa-let-7d-5p hsa-miR-4320 hsa-miR-6509-3p hsa-miR-

		379-3p hsa-miR-206 hsa-miR-4295 hsa-miR-6835-3p hsa-miR-25-3p hsa-miR-367-3p hsa-miR-3922-3p hsa-miR-6792-3p hsa-miR-4436b-5p hsa-miR-1343-3p hsa-miR-3166 hsa-miR-625-5p hsa-miR-216a-5p hsa-miR-6795-5p hsa-miR-6738-3p hsa-miR-130a-3p hsa-miR-5088-3p hsa-miR-500a-3p hsa-miR-5187-5p hsa-miR-138-2-3p hsa-miR-1254 hsa-miR-642b-3p hsa-miR-363-3p hsa-miR-7853-5p hsa-miR-3154 hsa-miR-22-5p hsa-miR-129-5p hsa-miR-6886-3p hsa-miR-411-3p hsa-miR-7111-3p hsa-miR-664b-3p hsa-miR-3925-5p hsa-miR-4458 hsa-miR-4761-3p hsa-miR-215-3p hsa-miR-449c-3p hsa-miR-8052 hsa-miR-5011-5p hsa-miR-92a-3p hsa-miR-4533 hsa-miR-4691-5p hsa-miR-98-5p hsa-miR-6083 hsa-miR-302d-5p hsa-miR-432-5p hsa-miR-5093 hsa-miR-4422 hsa-miR-526b-5p hsa-miR-3176 hsa-miR-1208 hsa-miR-6885-5p hsa-miR-6501-3p hsa-miR-1273d hsa-miR-3116 hsa-miR-4636 hsa-miR-596 hsa-miR-519d-5p hsa-miR-328-5p hsa-miR-8057 hsa-miR-7847-3p hsa-miR-6819-3p hsa-miR-6752-5p hsa-miR-3182 hsa-miR-3654 hsa-miR-4651 hsa-miR-7110-5p hsa-miR-6126 hsa-miR-6780a-3p hsa-miR-4704-3p hsa-let-7e-5p hsa-miR-301b-3p hsa-miR-105-5p hsa-miR-631 hsa-miR-4500 hsa-miR-7109-5p hsa-miR-5695 hsa-miR-3666 hsa-miR-5583-3p hsa-let-7a-5p hsa-miR-499a-3p hsa-miR-130b-3p hsa-let-7c-5p hsa-miR-6783-3p hsa-miR-6772-3p hsa-miR-6165 hsa-miR-454-3p hsa-miR-579-3p hsa-miR-6877-3p hsa-miR-7845-5p hsa-miR-4293 hsa-miR-6879-5p hsa-miR-4695-5p hsa-miR-613
SRC-2; SRC-3	203	hsa-miR-5197-3p hsa-miR-6738-5p hsa-miR-6874-3p hsa-miR-3940-5p hsa-miR-4314 hsa-miR-6515-3p hsa-miR-6499-3p hsa-miR-2276-3p hsa-miR-1306-5p hsa-miR-19b-1-5p hsa-miR-4747-5p hsa-miR-6732-5p hsa-miR-548ar-3p hsa-miR-4690-3p hsa-miR-6868-3p hsa-miR-6730-3p hsa-miR-4463 hsa-miR-6794-5p hsa-miR-135a-3p hsa-miR-653-5p hsa-miR-3162-3p hsa-miR-4722-5p hsa-miR-5196-5p hsa-miR-142-5p hsa-miR-766-3p hsa-miR-330-3p hsa-miR-4264 hsa-miR-10a-3p hsa-miR-3928-3p hsa-miR-769-5p hsa-miR-5706 hsa-miR-370-3p hsa-miR-3192-3p hsa-miR-6856-3p hsa-miR-4455 hsa-miR-3189-3p hsa-miR-6805-3p hsa-miR-4739 hsa-miR-576-5p hsa-miR-6504-3p hsa-miR-3925-3p hsa-miR-5585-5p hsa-miR-4804-3p hsa-miR-30a-3p hsa-miR-548d-3p hsa-miR-6733-3p hsa-miR-605-3p hsa-miR-3591-3p hsa-miR-7849-3p hsa-miR-548h-3p hsa-miR-183-3p hsa-miR-223-5p hsa-miR-1914-3p hsa-miR-3686 hsa-miR-4720-3p hsa-miR-3658 hsa-miR-21-3p hsa-miR-383-5p.1 hsa-miR-4666a-5p hsa-miR-656-3p hsa-miR-4782-5p hsa-miR-6839-3p hsa-miR-1323 hsa-miR-4521 hsa-miR-216b-5p hsa-miR-148b-5p hsa-miR-5197-5p hsa-miR-190a-3p hsa-miR-548bb-3p hsa-miR-6818-5p hsa-miR-3927-3p hsa-miR-6867-3p hsa-miR-2052 hsa-miR-6081 hsa-miR-4756-5p hsa-miR-6758-5p hsa-miR-1284 hsa-miR-4499 hsa-miR-1321 hsa-miR-548ax hsa-miR-520h hsa-miR-4713-5p hsa-miR-3607-5p hsa-miR-141-3p hsa-miR-6856-5p hsa-miR-186-5p hsa-miR-3915 hsa-miR-6750-5p hsa-miR-4803 hsa-miR-4772-3p hsa-miR-4251 hsa-miR-5195-3p hsa-miR-1972 hsa-miR-3120-5p hsa-miR-373-5p hsa-miR-4738-3p hsa-miR-423-5p hsa-miR-181c-5p hsa-miR-5685 hsa-miR-200a-3p hsa-miR-153-5p hsa-miR-548e-3p hsa-miR-548ac hsa-miR-6734-5p hsa-miR-219a-2-3p hsa-miR-505-3p.1 hsa-miR-891b hsa-miR-6792-5p hsa-miR-8059 hsa-miR-4520-2-3p hsa-miR-6077 hsa-miR-548o-3p hsa-miR-4789-3p hsa-miR-7702 hsa-miR-6824-5p hsa-miR-548f-3p hsa-miR-6831-5p hsa-miR-378j hsa-miR-6839-5p hsa-miR-4278 hsa-miR-3611 hsa-miR-4456 hsa-miR-5009-3p hsa-miR-3134 hsa-miR-3620-3p hsa-miR-5194 hsa-miR-5590-3p hsa-miR-181d-5p hsa-miR-616-5p hsa-miR-4733-5p hsa-miR-6805-5p hsa-miR-4494 hsa-miR-4645-3p hsa-miR-181b-5p hsa-miR-4760-3p hsa-miR-6893-3p hsa-miR-5589-3p hsa-miR-510-3p hsa-miR-3151-3p hsa-miR-4470 hsa-miR-5691 hsa-miR-6776-5p hsa-miR-5579-3p hsa-miR-6516-3p hsa-miR-151a-3p hsa-miR-4262 hsa-

		miR-582-3p hsa-miR-570-3p hsa-miR-520g-3p hsa-miR-3974 hsa-miR-7156-5p hsa-miR-325-3p hsa-miR-619-3p hsa-miR-4309 hsa-miR-3184-5p hsa-miR-664a-3p hsa-miR-767-3p hsa-miR-19a-5p hsa-miR-4529-3p hsa-miR-30e-3p hsa-miR-3198 hsa-miR-4471 hsa-miR-4772-5p hsa-miR-802 hsa-miR-3714 hsa-miR-545-5p hsa-miR-371b-5p hsa-miR-4668-3p hsa-miR-146a-3p hsa-miR-7151-3p hsa-miR-450b-5p hsa-miR-6783-5p hsa-miR-4716-3p hsa-miR-4495 hsa-miR-548a-3p hsa-miR-494-3p hsa-miR-19b-2-5p hsa-miR-451b hsa-miR-150-5p hsa-miR-3133 hsa-miR-8063 hsa-miR-203b-3p hsa-miR-4724-5p hsa-miR-30d-3p hsa-miR-4266 hsa-miR-4273 hsa-miR-219b-3p hsa-miR-181a-5p hsa-miR-6822-5p hsa-miR-629-3p hsa-miR-548z hsa-miR-577 hsa-miR-888-3p hsa-miR-4507 hsa-miR-548az-3p hsa-miR-1251-3p hsa-miR-548ao-5p hsa-miR-5095 hsa-miR-5582-3p hsa-miR-145-5p hsa-miR-3935 hsa-miR-4768-3p hsa-miR-512-5p
SRC-1	250	hsa-miR-4709-3p hsa-miR-6876-5p hsa-miR-3680-5p hsa-miR-367-5p hsa-miR-6760-3p hsa-miR-2278 hsa-miR-6759-3p hsa-miR-3688-5p hsa-miR-4677-3p hsa-miR-3122 hsa-miR-1248 hsa-miR-548b-5p hsa-miR-3074-5p hsa-miR-4525 hsa-miR-449a hsa-miR-4765 hsa-miR-6815-3p hsa-miR-6088 hsa-miR-4710 hsa-miR-6833-5p hsa-miR-191-3p hsa-miR-1915-3p hsa-miR-8073 hsa-miR-1301-3p hsa-miR-1273f hsa-miR-4491 hsa-miR-6751-5p hsa-miR-939-3p hsa-miR-548ad-5p hsa-miR-129-1-3p hsa-miR-6762-3p hsa-miR-548ae-5p hsa-miR-96-5p hsa-miR-5579-5p hsa-miR-4721 hsa-miR-17-3p hsa-miR-376a-3p hsa-miR-92a-1-5p hsa-miR-548am-5p hsa-miR-1255b-5p hsa-miR-670-3p hsa-miR-573 hsa-miR-4304 hsa-miR-148b-3p hsa-miR-3692-5p hsa-miR-8074 hsa-miR-513b-3p hsa-miR-152-3p hsa-miR-2277-3p hsa-miR-4277 hsa-miR-4496 hsa-miR-4679 hsa-miR-548d-5p hsa-miR-769-3p hsa-miR-143-3p hsa-miR-4323 hsa-miR-4665-5p hsa-miR-3129-5p hsa-miR-1343-5p hsa-miR-3960 hsa-miR-548aq-5p hsa-miR-4420 hsa-miR-6506-5p hsa-miR-676-3p hsa-miR-3142 hsa-miR-7106-5p hsa-miR-376b-3p hsa-miR-491-3p hsa-miR-4659a-5p hsa-miR-4778-3p hsa-miR-376c-3p hsa-miR-4758-3p hsa-miR-1263 hsa-miR-670-5p hsa-miR-4659b-5p hsa-miR-1914-5p hsa-miR-181b-3p hsa-miR-5047 hsa-miR-6829-5p hsa-miR-492 hsa-miR-3175 hsa-miR-3158-5p hsa-miR-4742-5p hsa-miR-4476 hsa-miR-5580-5p hsa-miR-4705 hsa-miR-2116-5p hsa-miR-6840-3p hsa-miR-1271-5p hsa-miR-548j-5p hsa-miR-548ay-5p hsa-miR-3936 hsa-miR-4799-3p hsa-miR-2053 hsa-miR-1236-5p hsa-miR-487a-5p hsa-miR-548au-3p hsa-miR-939-5p hsa-miR-8072 hsa-miR-5010-5p hsa-miR-4684-3p hsa-miR-6728-5p hsa-miR-548o-5p hsa-miR-3688-3p hsa-miR-1265 hsa-miR-5694 hsa-miR-3659 hsa-miR-6882-5p hsa-miR-4694-3p hsa-miR-548h-5p hsa-miR-1275 hsa-miR-22-3p hsa-miR-1205 hsa-miR-34c-5p hsa-miR-889-5p hsa-miR-3619-3p hsa-miR-3127-5p hsa-miR-6503-3p hsa-miR-4284 hsa-miR-1184 hsa-miR-517-5p hsa-miR-4635 hsa-miR-4797-5p hsa-miR-604 hsa-miR-181b-2-3p hsa-miR-493-3p hsa-miR-6763-5p hsa-miR-890 hsa-miR-548a-5p hsa-miR-647 hsa-miR-4726-3p hsa-miR-559 hsa-miR-127-5p hsa-miR-6869-5p hsa-miR-383-5p.2 hsa-miR-6776-3p hsa-miR-24-3p hsa-miR-1238-3p hsa-miR-3190-3p hsa-miR-8068 hsa-miR-138-5p hsa-miR-6852-3p hsa-miR-6816-3p hsa-miR-658 hsa-miR-182-5p hsa-miR-1245b-5p hsa-miR-548ak hsa-miR-5588-5p hsa-miR-2467-5p hsa-miR-6872-3p hsa-miR-4439 hsa-miR-548c-5p hsa-miR-514a-3p hsa-miR-3909 hsa-miR-5585-3p hsa-miR-1911-3p hsa-miR-4417 hsa-miR-548ap-5p hsa-miR-4637 hsa-miR-653-3p hsa-miR-299-5p hsa-miR-6803-5p hsa-miR-7977 hsa-miR-5581-3p hsa-miR-10b-3p hsa-miR-6513-5p hsa-miR-5100 hsa-miR-548ar-5p hsa-miR-1305 hsa-miR-6852-5p hsa-miR-6870-3p hsa-miR-548i hsa-miR-4483 hsa-miR-148a-3p hsa-miR-6851-3p hsa-miR-129-2-3p hsa-miR-7156-3p hsa-miR-5001-3p hsa-miR-1273h-3p hsa-miR-2115-3p

		hsa-miR-7152-5p hsa-miR-7855-5p hsa-miR-548as-5p hsa-miR-4764-5p hsa-miR-134-3p hsa-miR-936 hsa-miR-2861 hsa-miR-5190 hsa-miR-6835-5p hsa-miR-496.1 hsa-miR-449b-5p hsa-miR-548w hsa-miR-3913-5p hsa-miR-3616-5p hsa-miR-7107-3p hsa-miR-93-3p hsa-miR-450b-3p hsa-miR-6894-5p hsa-miR-4792 hsa-miR-6753-3p hsa-miR-299-3p hsa-miR-25-5p hsa-miR-6823-5p hsa-miR-548bb-5p hsa-miR-1255a hsa-miR-3973 hsa-miR-548au-5p hsa-miR-4727-5p hsa-miR-221-5p hsa-miR-891a-3p hsa-miR-619-5p hsa-miR-605-5p hsa-miR-4720-5p hsa-miR-5684 hsa-miR-4446-3p hsa-miR-4298 hsa-miR-4776-5p hsa-miR-199a-3p hsa-miR-4769-3p hsa-miR-6764-5p hsa-miR-548ab hsa-miR-548y hsa-miR-34a-5p hsa-miR-4425 hsa-miR-7843-3p hsa-miR-6728-3p hsa-miR-3136-5p hsa-miR-4771 hsa-miR-199b-3p hsa-miR-3150a-3p hsa-miR-4770 hsa-miR-4658 hsa-miR-6071 hsa-miR-6742-3p hsa-miR-6790-5p hsa-miR-3614-3p hsa-miR-574-5p hsa-miR-4657 hsa-miR-296-3p hsa-miR-877-3p hsa-miR-6087 hsa-miR-487b-5p hsa-miR-649 hsa-miR-205-3p hsa-miR-4800-5p hsa-miR-6817-5p hsa-miR-1302 hsa-miR-514b-3p hsa-miR-1293 hsa-miR-4502
SRC-2	354	hsa-miR-576-3p hsa-miR-2682-5p hsa-miR-487a-3p hsa-miR-34b-5p hsa-miR-381-3p hsa-miR-374b-3p hsa-miR-3681-5p hsa-miR-548s hsa-miR-3187-3p hsa-miR-3155a hsa-miR-4328 hsa-miR-2114-3p hsa-miR-3120-3p hsa-miR-138-1-3p hsa-miR-675-3p hsa-miR-4732-5p hsa-miR-7162-5p hsa-miR-8060 hsa-miR-4421 hsa-miR-4486 hsa-miR-1179 hsa-miR-3672 hsa-let-7a-3p hsa-miR-532-3p hsa-miR-3653-5p hsa-miR-202-5p hsa-miR-516a-3p hsa-miR-575 hsa-miR-6515-5p hsa-miR-375 hsa-miR-627-5p hsa-miR-3618 hsa-miR-664b-5p hsa-miR-302c-5p hsa-miR-3202 hsa-miR-124-3p.1 hsa-miR-3910 hsa-let-7b-3p hsa-miR-6878-5p hsa-miR-885-5p hsa-miR-4715-3p hsa-miR-6799-5p hsa-miR-3677-5p hsa-miR-7848-3p hsa-miR-342-3p hsa-miR-5587-5p hsa-miR-4326 hsa-miR-1231 hsa-miR-199a-5p hsa-miR-5680 hsa-miR-3682-3p hsa-miR-657 hsa-miR-9500 hsa-miR-136-3p hsa-miR-876-5p hsa-miR-4419b hsa-miR-7150 hsa-miR-3689a-5p hsa-miR-1228-3p hsa-miR-4744 hsa-miR-5006-5p hsa-miR-3678-3p hsa-miR-4711-3p hsa-miR-503-3p hsa-miR-3680-3p hsa-miR-7112-5p hsa-miR-6772-5p hsa-miR-4680-5p hsa-miR-504-5p.1 hsa-miR-4678 hsa-miR-5708 hsa-miR-660-3p hsa-miR-1202 hsa-miR-3160-5p hsa-miR-6715b-3p hsa-miR-6864-3p hsa-miR-216b-3p hsa-miR-6837-3p hsa-miR-4263 hsa-miR-214-5p hsa-miR-495-5p hsa-miR-643 hsa-miR-4735-5p hsa-miR-3162-5p hsa-miR-6893-5p hsa-miR-4539 hsa-miR-4717-3p hsa-miR-6854-5p hsa-miR-509-3p hsa-miR-4722-3p hsa-miR-124-3p.2 hsa-miR-4292 hsa-miR-4650-5p hsa-miR-3617-3p hsa-miR-2681-5p hsa-miR-3613-5p hsa-miR-5096 hsa-miR-6808-5p hsa-miR-6837-5p hsa-miR-98-3p hsa-miR-6743-5p hsa-miR-154-3p hsa-miR-4774-3p hsa-miR-516b-3p hsa-miR-7705 hsa-miR-3972 hsa-miR-153-3p hsa-miR-331-5p hsa-miR-31-5p hsa-miR-3924 hsa-miR-6508-3p hsa-miR-5193 hsa-miR-139-3p hsa-miR-651-5p hsa-miR-6826-3p hsa-miR-2681-3p hsa-miR-6858-3p hsa-miR-6073 hsa-miR-4711-5p hsa-miR-6730-5p hsa-miR-3650 hsa-miR-4789-5p hsa-miR-4686 hsa-miR-208a-5p hsa-miR-4478 hsa-miR-4307 hsa-miR-139-5p hsa-miR-301a-5p hsa-miR-155-3p hsa-miR-103a-3p hsa-miR-383-3p hsa-miR-199b-5p hsa-miR-8062 hsa-miR-8079 hsa-miR-759 hsa-miR-1915-5p hsa-miR-6789-5p hsa-miR-4745-5p hsa-miR-6511b-5p hsa-miR-144-3p hsa-miR-374a-3p hsa-miR-7515 hsa-miR-5007-3p hsa-miR-6823-3p hsa-miR-218-5p hsa-miR-6820-3p hsa-miR-208b-5p hsa-miR-3064-3p hsa-miR-136-5p hsa-miR-3689e hsa-miR-3689f hsa-miR-4272 hsa-miR-632 hsa-miR-101-3p.1 hsa-miR-556-3p hsa-miR-4289 hsa-miR-3668 hsa-miR-499b-5p hsa-miR-3184-3p hsa-miR-29b-1-5p hsa-miR-4526 hsa-miR-3164 hsa-miR-6514-3p hsa-miR-4736 hsa-miR-140-3p.2 hsa-miR-4474-5p hsa-miR-1537-3p hsa-miR-6884-3p hsa-miR-3606-5p

		hsa-miR-3147 hsa-miR-5700 hsa-miR-3152-5p hsa-miR-4275 hsa-miR-4754 hsa-miR-4306 hsa-miR-3689b-5p hsa-miR-3918 hsa-miR-185-5p hsa-miR-888-5p hsa-miR-181c-3p hsa-miR-624-3p hsa-miR-6890-5p hsa-miR-875-5p hsa-miR-942-3p hsa-miR-1245a hsa-miR-3118 hsa-miR-4531 hsa-miR-6892-3p hsa-miR-1290 hsa-miR-7154-5p hsa-miR-6513-3p hsa-miR-28-3p hsa-miR-4640-5p hsa-miR-196a-5p hsa-miR-6516-5p hsa-miR-3146 hsa-miR-510-5p hsa-miR-4726-5p hsa-miR-4644 hsa-miR-301b-5p hsa-miR-6716-5p hsa-miR-6889-5p hsa-miR-589-3p hsa-miR-4685-5p hsa-miR-6853-3p hsa-miR-6780b-3p hsa-miR-4528 hsa-miR-2116-3p hsa-miR-6895-5p hsa-miR-134-5p hsa-miR-7157-3p hsa-miR-323a-3p hsa-miR-3065-3p hsa-miR-3143 hsa-miR-196b-5p hsa-miR-6509-5p hsa-miR-6791-5p hsa-miR-6850-5p hsa-miR-6889-3p hsa-miR-587 hsa-miR-1303 hsa-miR-1304-5p hsa-miR-142-3p.1 hsa-miR-8087 hsa-miR-3137 hsa-miR-1185-2-3p hsa-miR-4794 hsa-miR-3685 hsa-miR-6727-3p hsa-miR-1273e hsa-miR-337-3p hsa-miR-660-5p hsa-miR-3660 hsa-miR-378g hsa-miR-6894-3p hsa-miR-521 hsa-miR-3155b hsa-miR-590-5p hsa-miR-106b-3p hsa-miR-2113 hsa-miR-6736-3p hsa-miR-942-5p hsa-miR-622 hsa-miR-6739-3p hsa-miR-3177-5p hsa-miR-6760-5p hsa-miR-506-3p hsa-miR-6811-5p hsa-miR-31-3p hsa-miR-7113-3p hsa-miR-203a-3p.1 hsa-miR-1286 hsa-miR-101-3p.2 hsa-miR-873-5p.2 hsa-miR-3675-5p hsa-miR-484 hsa-miR-4701-5p hsa-miR-6777-5p hsa-miR-204-3p hsa-miR-96-3p hsa-miR-4676-5p hsa-miR-4688 hsa-miR-6865-3p hsa-miR-1178-3p hsa-miR-3944-5p hsa-miR-7852-3p hsa-miR-34c-3p hsa-miR-1253 hsa-miR-4646-5p hsa-miR-520f-5p hsa-miR-4712-3p hsa-miR-4481 hsa-miR-20b-3p hsa-miR-5188 hsa-miR-6719-3p hsa-miR-2115-5p hsa-miR-4662a-5p hsa-miR-561-5p hsa-miR-3158-3p hsa-miR-6753-5p hsa-miR-940 hsa-miR-4653-5p hsa-miR-588 hsa-miR-1261 hsa-miR-1251-5p hsa-miR-449c-5p hsa-miR-5087 hsa-miR-6735-3p hsa-miR-548av-3p hsa-miR-5701 hsa-miR-4704-5p hsa-miR-3152-3p hsa-miR-6834-5p hsa-miR-934 hsa-miR-548b-3p hsa-miR-1322 hsa-miR-148a-5p hsa-miR-609 hsa-miR-4440 hsa-let-7f-2-3p hsa-miR-3167 hsa-miR-4660 hsa-miR-1292-5p hsa-miR-3144-3p hsa-let-7f-1-3p hsa-miR-100-3p hsa-miR-1227-3p hsa-miR-6843-3p hsa-miR-3929 hsa-miR-1256 hsa-miR-4755-3p hsa-miR-3921 hsa-miR-6737-3p hsa-miR-4435 hsa-miR-664a-5p hsa-miR-466 hsa-miR-6848-3p hsa-miR-4288 hsa-miR-6747-3p hsa-miR-374c-3p hsa-miR-1264 hsa-miR-6830-3p hsa-miR-5699-3p hsa-miR-382-5p hsa-miR-6849-5p hsa-miR-107 hsa-miR-5003-5p hsa-miR-5010-3p hsa-miR-300 hsa-miR-4733-3p hsa-miR-4475 hsa-miR-3940-3p hsa-miR-218-2-3p hsa-miR-212-5p hsa-miR-889-3p hsa-miR-15a-3p hsa-miR-1226-5p hsa-miR-3665 hsa-miR-4423-3p hsa-miR-5008-3p hsa-miR-6752-3p hsa-miR-346 hsa-miR-21-5p hsa-miR-4786-5p hsa-miR-1185-1-3p hsa-miR-6846-3p hsa-miR-8065 hsa-miR-4252 hsa-miR-552-5p hsa-miR-3159 hsa-miR-433-3p hsa-miR-6830-5p hsa-miR-1976 hsa-miR-4687-5p
SRC-3	354	hsa-miR-520f-3p hsa-miR-1298-5p hsa-miR-30d-5p hsa-miR-4493 hsa-miR-520b hsa-miR-7159-5p hsa-miR-4524b-3p hsa-miR-17-5p hsa-miR-6895-3p hsa-miR-6873-5p hsa-miR-3200-5p hsa-miR-302b-3p hsa-miR-5089-3p hsa-miR-648 hsa-miR-4695-3p hsa-miR-6793-3p hsa-miR-541-5p hsa-miR-1276 hsa-miR-519b-3p hsa-miR-4641 hsa-miR-193a-5p hsa-miR-4534 hsa-miR-4474-3p hsa-miR-500a-5p hsa-miR-758-3p hsa-miR-4274 hsa-miR-3150b-3p hsa-miR-6858-5p hsa-miR-4797-3p hsa-miR-4650-3p hsa-miR-593-3p hsa-miR-320c hsa-miR-6818-3p hsa-miR-6504-5p hsa-miR-4708-3p hsa-miR-4748 hsa-miR-4302 hsa-miR-4436a hsa-miR-486-3p hsa-miR-6511a-3p hsa-miR-5583-5p hsa-miR-3619-5p hsa-miR-3689d hsa-miR-200a-5p hsa-miR-302e hsa-miR-302a-5p hsa-miR-7155-5p hsa-miR-544b hsa-miR-4633-3p hsa-miR-3938 hsa-miR-7112-3p hsa-miR-938 hsa-miR-3074-3p hsa-miR-329-5p hsa-miR-372-3p hsa-miR-30a-5p hsa-miR-

		6844 hsa-miR-623 hsa-miR-4689 hsa-miR-519a-3p hsa-miR-621 hsa-miR-4664-5p hsa-miR-4291 hsa-miR-1271-3p hsa-miR-496.2 hsa-miR-491-5p hsa-miR-302c-3p.2 hsa-miR-3670 hsa-miR-4286 hsa-miR-6887-3p hsa-miR-196a-3p hsa-miR-3153 hsa-miR-548ba hsa-miR-8080 hsa-miR-1912 hsa-miR-4316 hsa-miR-302c-3p.1 hsa-miR-203a-5p hsa-miR-4717-5p hsa-miR-20b-5p hsa-miR-4297 hsa-miR-3065-5p hsa-miR-6822-3p hsa-miR-323b-3p hsa-miR-4784 hsa-miR-4418 hsa-miR-4640-3p hsa-miR-106b-5p hsa-miR-4712-5p hsa-miR-504-3p hsa-miR-379-5p hsa-miR-6069 hsa-miR-4511 hsa-miR-580-5p hsa-miR-20a-5p hsa-miR-106a-5p hsa-miR-188-3p hsa-miR-550b-2-5p hsa-miR-6859-5p hsa-miR-2117 hsa-miR-548u hsa-miR-6801-5p hsa-miR-4638-3p hsa-miR-6808-3p hsa-miR-141-5p hsa-miR-4676-3p hsa-miR-4756-3p hsa-miR-6871-5p hsa-miR-6821-3p hsa-miR-520c-3p hsa-miR-518a-5p hsa-miR-770-5p hsa-miR-6817-3p hsa-miR-200b-5p hsa-miR-3157-3p hsa-miR-5195-5p hsa-miR-4280 hsa-miR-1294 hsa-miR-7158-5p hsa-miR-3168 hsa-miR-6874-5p hsa-miR-302a-3p hsa-miR-4299 hsa-miR-512-3p hsa-miR-7110-3p hsa-miR-4536-5p hsa-miR-4760-5p hsa-miR-935 hsa-miR-541-3p hsa-miR-4503 hsa-miR-4999-3p hsa-miR-497-3p hsa-miR-8082 hsa-miR-4662b hsa-miR-371a-5p hsa-miR-4642 hsa-miR-4724-3p hsa-miR-4524a-3p hsa-miR-30b-5p hsa-miR-4429 hsa-miR-330-5p hsa-miR-571 hsa-miR-3160-3p hsa-miR-548ag hsa-miR-7152-3p hsa-miR-3125 hsa-miR-342-5p hsa-miR-4696 hsa-miR-527 hsa-miR-224-5p hsa-miR-29c-3p hsa-miR-3616-3p hsa-miR-4311 hsa-miR-30e-5p hsa-miR-7161-5p hsa-miR-411-5p.2 hsa-miR-3117-5p hsa-miR-1279 hsa-miR-93-5p hsa-miR-4452 hsa-miR-654-5p hsa-miR-6866-3p hsa-miR-6511b-3p hsa-miR-7158-3p hsa-miR-6734-3p hsa-miR-6068 hsa-miR-24-1-5p hsa-miR-5584-3p hsa-miR-4424 hsa-miR-6812-5p hsa-miR-6851-5p hsa-miR-876-3p hsa-miR-211-5p hsa-miR-509-5p hsa-miR-767-5p hsa-miR-219a-1-3p hsa-miR-6134 hsa-miR-132-3p hsa-miR-514a-5p hsa-miR-3190-5p hsa-miR-6756-5p hsa-miR-595 hsa-miR-7854-3p hsa-miR-552-3p hsa-miR-570-5p hsa-miR-4646-3p hsa-miR-3529-5p hsa-miR-1285-5p hsa-miR-3919 hsa-miR-3916 hsa-miR-3064-5p hsa-miR-509-3-5p hsa-miR-522-3p hsa-miR-373-3p hsa-miR-4755-5p hsa-miR-500b-5p hsa-miR-6807-3p hsa-miR-922 hsa-miR-6766-5p hsa-miR-5581-5p hsa-miR-411-5p.1 hsa-miR-4778-5p hsa-miR-892c-3p hsa-miR-6507-3p hsa-miR-6130 hsa-miR-3907 hsa-miR-561-3p hsa-miR-6512-5p hsa-miR-526b-3p hsa-miR-4426 hsa-miR-1273g-5p hsa-miR-4663 hsa-miR-4419a hsa-miR-520a-3p hsa-miR-4520-3p hsa-miR-6888-3p hsa-miR-629-5p hsa-miR-6733-5p hsa-miR-625-3p hsa-miR-6861-5p hsa-miR-8061 hsa-miR-519c-3p hsa-miR-4487 hsa-miR-1295b-3p hsa-miR-217 hsa-miR-892b hsa-miR-7159-3p hsa-miR-3136-3p hsa-miR-1288-3p hsa-miR-4672 hsa-miR-15b-3p hsa-miR-3934-3p hsa-miR-30c-5p hsa-miR-4649-3p hsa-miR-6804-3p hsa-miR-193b-5p hsa-miR-7155-3p hsa-miR-550a-5p hsa-miR-6129 hsa-miR-548p hsa-miR-4687-3p hsa-miR-519d-3p hsa-miR-6806-5p hsa-miR-29b-3p hsa-miR-4451 hsa-miR-7974 hsa-miR-6768-5p hsa-miR-302f hsa-miR-550b-3p hsa-miR-606 hsa-miR-1178-5p hsa-miR-518c-5p hsa-miR-1468-5p hsa-miR-5192 hsa-miR-4433a-3p hsa-miR-4324 hsa-miR-3135a hsa-miR-5571-3p hsa-miR-4779 hsa-miR-3145-3p hsa-miR-676-5p hsa-miR-29a-3p hsa-miR-550a-3-5p hsa-miR-3663-3p hsa-miR-6831-3p hsa-miR-320d hsa-miR-6824-3p hsa-miR-4464 hsa-miR-6769b-5p hsa-miR-1277-3p hsa-miR-1243 hsa-miR-761 hsa-miR-149-5p hsa-miR-4318 hsa-miR-4490 hsa-miR-616-3p hsa-miR-212-3p hsa-miR-6764-3p hsa-miR-505-3p.2 hsa-miR-4468 hsa-miR-224-3p hsa-miR-520d-3p hsa-miR-6747-5p hsa-miR-4790-3p hsa-miR-452-5p hsa-miR-6737-5p hsa-miR-642a-5p hsa-miR-3156-5p hsa-miR-520e hsa-miR-580-3p hsa-miR-6739-5p hsa-miR-3667-3p hsa-miR-4716-5p hsa-miR-7975 hsa-miR-1295b-5p hsa-miR-6838-3p hsa-miR-215-5p hsa-

		miR-6127 hsa-miR-6758-3p hsa-miR-6133 hsa-miR-4510 hsa-miR-583 hsa-miR-760 hsa-miR-6777-3p hsa-miR-323a-5p hsa-miR-877-5p hsa-miR-4700-3p hsa-miR-5000-3p hsa-miR-92a-2-5p hsa-miR-1224-3p hsa-miR-4774-5p hsa-miR-6798-3p hsa-miR-1910-5p hsa-miR-3914 hsa-miR-6769a-5p hsa-miR-320a hsa-miR-1225-5p hsa-miR-6819-5p hsa-miR-6749-3p hsa-miR-4647 hsa-miR-7162-3p hsa-miR-3130-3p hsa-miR-6131 hsa-miR-320b hsa-miR-505-5p hsa-miR-4729 hsa-miR-140-3p.1 hsa-miR-3664-5p hsa-miR-192-5p hsa-miR-6080 hsa-miR-6729-3p hsa-miR-5689 hsa-miR-5006-3p hsa-miR-874-3p hsa-miR-3129-3p hsa-miR-376a-5p hsa-miR-188-5p hsa-miR-7978 hsa-miR-6873-3p hsa-miR-4671-3p hsa-miR-204-5p hsa-miR-2054 hsa-miR-548ai hsa-miR-7973 hsa-miR-214-3p hsa-miR-1197 hsa-miR-197-3p hsa-miR-326 hsa-miR-24-2-5p hsa-miR-4780 hsa-miR-2355-5p hsa-miR-6500-5p hsa-miR-558 hsa-miR-302d-3p

Table S1. Binding regions between miR-137 and its putative target genes, SRC-1, SRC-2, and SRC-3 (miRDB).

Names	Total	microRNAs
SRC-1; SRC-2; SRC-3	11	hsa-miR-8485 hsa-miR-1277-5p hsa-miR-3191-5p hsa-miR-548az-5p hsa-miR-4699-3p hsa-miR-137-3p hsa-miR-3646 hsa-miR-548t-5p hsa-miR-548c-3p hsa-miR-1283.
SRC-1; SRC-2	32	hsa-miR-23a-3p hsa-miR-513a-3p hsa-miR-4443 hsa-miR-506-5p hsa-miR-548t-3p hsa-miR-3606-3p hsa-miR-4441 hsa-miR-590-3p hsa-miR-10522-5p hsa-miR-23c hsa-miR-4786-3p hsa-miR-23b-3p hsa-miR-548aa hsa-miR-452-3p hsa-miR-4719 hsa-miR-548ap-3p hsa-miR-4670-3p hsa-miR-501-5p hsa-miR-4270 hsa-miR-2467-3p hsa-miR-4279 hsa-miR-6888-5p hsa-miR-4775 hsa-miR-4666a-3p hsa-miR-513c-3p hsa-miR-3679-3p hsa-miR-5580-3p hsa-miR-369-3p hsa-miR-548as-3p hsa-miR-4428 hsa-miR-6809-3p.
SRC-1; SRC-3	25	hsa-miR-489-3p hsa-miR-514b-5p hsa-miR-655-3p hsa-miR-875-3p hsa-miR-524-5p hsa-miR-211-3p hsa-miR-5011-5p hsa-miR-4652-3p hsa-miR-4482-3p hsa-miR-4422 hsa-miR-6875-3p hsa-miR-5004-3p hsa-miR-545-3p hsa-miR-4254 hsa-miR-6882-3p hsa-miR-548e-5p hsa-miR-374c-5p hsa-miR-9985 hsa-miR-6507-5p hsa-miR-544a hsa-miR-27b-3p hsa-miR-513c-5p hsa-miR-500a-3p hsa-miR-520d-5p hsa-miR-27a-3p.
SRC-2; SRC-3	25	hsa-miR-7-2-3p hsa-miR-9-5p hsa-miR-7-1-3p hsa-miR-3662 hsa-miR-3613-3p hsa-miR-548m hsa-miR-766-3p hsa-miR-5787 hsa-miR-510-3p hsa-miR-200c-3p hsa-miR-200b-3p hsa-miR-3925-3p hsa-miR-12136 hsa-miR-4762-3p hsa-miR-520g-3p hsa-miR-6839-3p hsa-miR-216b-5p hsa-miR-429 hsa-miR-4668-3p hsa-miR-3913-3p hsa-miR-520h hsa-miR-186-5p hsa-miR-4738-3p hsa-miR-5582-3p hsa-miR-1252-3p.
SRC-1	137	hsa-miR-140-5p hsa-miR-3622b-3p hsa-miR-889-5p hsa-miR-5698 hsa-miR-222-5p hsa-miR-103a-2-5p hsa-miR-377-5p hsa-miR-650 hsa-miR-3622a-3p hsa-miR-3681-3p hsa-miR-4635 hsa-miR-18b-5p hsa-miR-18a-5p hsa-miR-216a-3p hsa-miR-374a-5p hsa-miR-2278 hsa-miR-892c-5p hsa-miR-493-3p hsa-miR-34a-3p hsa-miR-647 hsa-miR-4668-5p hsa-miR-496 hsa-miR-7853-5p hsa-miR-661 hsa-miR-493-5p hsa-miR-3074-5p hsa-miR-6825-5p hsa-miR-4735-3p hsa-miR-1238-3p hsa-miR-3908 hsa-miR-5584-5p hsa-miR-6815-3p hsa-miR-1-3p hsa-miR-129-5p hsa-miR-3942-5p hsa-miR-182-5p hsa-miR-103a-1-5p hsa-miR-6886-3p hsa-miR-551b-5p hsa-miR-1915-3p hsa-miR-3612 hsa-miR-6804-3p hsa-miR-653-3p hsa-miR-10b-3p hsa-miR-12123 hsa-miR-548ay-3p hsa-miR-6852-5p hsa-miR-320e hsa-miR-26b-3p hsa-miR-148a-3p hsa-miR-5692c hsa-miR-5001-3p hsa-miR-1273h-3p hsa-miR-2115-3p hsa-miR-7152-5p hsa-miR-1255b-5p hsa-miR-670-3p hsa-miR-5093 hsa-miR-6128 hsa-miR-6782-5p hsa-miR-148b-3p hsa-miR-301a-3p hsa-miR-936 hsa-miR-152-3p hsa-miR-1233-3p hsa-miR-2861 hsa-miR-2277-3p hsa-miR-6757-3p hsa-miR-708-3p hsa-miR-374b-5p hsa-miR-4509 hsa-miR-9983-3p hsa-miR-508-3p hsa-miR-519d-5p hsa-miR-3059-5p hsa-miR-93-3p hsa-miR-3129-5p hsa-miR-7847-3p hsa-miR-3182 hsa-miR-5692b hsa-miR-488-5p hsa-miR-4778-3p hsa-miR-376c-3p hsa-miR-1255a hsa-miR-1468-3p hsa-miR-12128 hsa-miR-6780a-3p hsa-miR-4728-3p hsa-miR-548at-3p hsa-miR-1914-5p hsa-miR-4704-3p hsa-miR-128-3p hsa-miR-4708-5p hsa-miR-7151-5p hsa-miR-6757-5p hsa-miR-4703-5p hsa-miR-7114-5p hsa-miR-301b-3p hsa-miR-105-5p hsa-miR-199a-3p hsa-miR-6764-5p hsa-miR-7111-5p hsa-miR-7843-3p hsa-miR-4766-3p hsa-miR-1299 hsa-miR-3666 hsa-miR-3922-5p hsa-miR-2053 hsa-miR-4330 hsa-miR-130b-3p hsa-miR-377-3p hsa-miR-6783-3p hsa-miR-6772-3p hsa-miR-199b-3p hsa-miR-6165 hsa-miR-454-3p hsa-miR-1250-3p hsa-miR-206 hsa-miR-4295 hsa-miR-6728-5p hsa-miR-6071 hsa-miR-6870-5p hsa-miR-3614-3p hsa-miR-3121-5p hsa-miR-1343-3p hsa-miR-3166 hsa-miR-877-3p hsa-miR-6738-3p hsa-miR-6086

Names	Total	microRNAs
		hsa-miR-130a-3p hsa-miR-498-5p hsa-miR-22-3p hsa-miR-6124 hsa-miR-613 hsa-miR-4723-5p hsa-miR-4653-3p hsa-miR-1225-3p.
SRC-2	223	hsa-miR-8081 hsa-miR-5197-3p hsa-miR-522-5p hsa-miR-518d-5p hsa-miR-381-3p hsa-miR-4306 hsa-miR-3918 hsa-miR-365b-5p hsa-miR-185-5p hsa-miR-873-5p hsa-miR-448 hsa-miR-4477b hsa-miR-3940-5p hsa-miR-4768-5p hsa-miR-548o-3p hsa-miR-4777-3p hsa-miR-6529-5p hsa-miR-335-3p hsa-miR-875-5p hsa-miR-1245a hsa-miR-1290 hsa-miR-7154-5p hsa-miR-6780b-5p hsa-miR-6831-5p hsa-miR-4659a-3p hsa-miR-3611 hsa-miR-1179 hsa-let-7a-3p hsa-miR-10527-5p hsa-miR-3146 hsa-miR-4325 hsa-miR-4644 hsa-miR-4463 hsa-miR-3134 hsa-miR-518e-5p hsa-miR-589-3p hsa-miR-597-3p hsa-miR-4477a hsa-miR-4722-5p hsa-miR-627-5p hsa-miR-4685-5p hsa-miR-3618 hsa-miR-181d-5p hsa-miR-6508-5p hsa-miR-2116-3p hsa-miR-302c-5p hsa-miR-507 hsa-miR-7113-5p hsa-miR-330-3p hsa-miR-4430 hsa-miR-365a-5p hsa-miR-548n hsa-let-7b-3p hsa-miR-3910 hsa-miR-6878-5p hsa-miR-3143 hsa-miR-4494 hsa-miR-4505 hsa-miR-181b-5p hsa-miR-4777-5p hsa-miR-6509-5p hsa-miR-342-3p hsa-miR-5587-5p hsa-miR-8055 hsa-miR-1231 hsa-miR-587 hsa-miR-5680 hsa-miR-4455 hsa-miR-4438 hsa-miR-6880-5p hsa-miR-5003-3p hsa-miR-576-5p hsa-miR-3137 hsa-miR-3692-3p hsa-miR-4659b-3p hsa-miR-5579-3p hsa-miR-4282 hsa-miR-627-3p hsa-miR-5585-5p hsa-miR-4804-3p hsa-miR-3941 hsa-miR-548d-3p hsa-miR-590-5p hsa-miR-523-5p hsa-miR-4262 hsa-miR-4725-3p hsa-miR-3148 hsa-miR-2113 hsa-miR-942-5p hsa-miR-548h-3p hsa-miR-6739-3p hsa-miR-4457 hsa-miR-1207-3p hsa-miR-6760-5p hsa-miR-3680-3p hsa-miR-4680-3p hsa-miR-4720-3p hsa-miR-3160-5p hsa-miR-12135 hsa-miR-3658 hsa-miR-6837-3p hsa-miR-214-5p hsa-miR-383-5p hsa-miR-526a-5p hsa-miR-495-5p hsa-miR-4735-5p hsa-miR-6833-3p hsa-miR-656-3p hsa-miR-520f-5p hsa-miR-6750-3p hsa-miR-5681b hsa-miR-1323 hsa-miR-4521 hsa-miR-561-5p hsa-miR-568 hsa-miR-654-3p hsa-miR-802 hsa-miR-6837-5p hsa-miR-98-3p hsa-miR-1261 hsa-miR-146a-3p hsa-miR-7151-3p hsa-miR-135b-5p hsa-miR-651-3p hsa-miR-125a-3p hsa-miR-548av-3p hsa-miR-7705 hsa-miR-153-3p hsa-miR-5197-5p hsa-miR-4704-5p hsa-miR-4271 hsa-miR-31-5p hsa-miR-3924 hsa-miR-520c-5p hsa-miR-548bb-3p hsa-miR-6818-5p hsa-miR-4766-5p hsa-miR-6782-3p hsa-miR-548b-3p hsa-miR-10397-3p hsa-miR-6754-5p hsa-miR-1322 hsa-miR-6842-3p hsa-miR-148a-5p hsa-miR-1257 hsa-miR-6073 hsa-miR-6730-5p hsa-miR-494-3p hsa-miR-4789-5p hsa-miR-4686 hsa-miR-208a-5p hsa-miR-4307 hsa-miR-451b hsa-miR-937-5p hsa-miR-7856-5p hsa-miR-361-3p hsa-miR-3128 hsa-let-7f-1-3p hsa-miR-5702 hsa-miR-383-3p hsa-miR-1284 hsa-miR-8079 hsa-miR-3185 hsa-miR-4499 hsa-miR-519a-5p hsa-miR-12129 hsa-miR-32-3p hsa-miR-1915-5p hsa-miR-4266 hsa-miR-335-5p hsa-miR-4288 hsa-miR-141-3p hsa-miR-181a-5p hsa-miR-7515 hsa-miR-374c-3p hsa-miR-135a-5p hsa-miR-1264 hsa-miR-208b-5p hsa-miR-518f-5p hsa-miR-9851-5p hsa-miR-6830-3p hsa-miR-8067 hsa-miR-196a-1-3p hsa-miR-629-3p hsa-miR-548z hsa-miR-577 hsa-miR-382-5p hsa-miR-136-5p hsa-miR-218-1-3p hsa-miR-7703 hsa-miR-5010-3p hsa-miR-4804-5p hsa-miR-300 hsa-miR-3120-5p hsa-miR-4507 hsa-miR-4475 hsa-miR-152-5p hsa-miR-181c-5p hsa-miR-3920 hsa-miR-519b-5p hsa-miR-499b-5p hsa-miR-200a-3p hsa-miR-153-5p hsa-miR-944 hsa-miR-548ac hsa-miR-548g-3p hsa-miR-1251-3p hsa-miR-655-5p hsa-miR-519c-5p hsa-miR-6752-3p hsa-miR-21-5p hsa-miR-6885-3p hsa-miR-6734-5p hsa-miR-6514-3p hsa-miR-8065 hsa-miR-3652 hsa-miR-4768-3p hsa-miR-433-3p hsa-miR-891b hsa-miR-9898 hsa-miR-6830-5p hsa-miR-539-5p hsa-miR-3124-3p.
SRC-3	144	hsa-miR-1298-5p hsa-miR-30d-5p hsa-miR-3916 hsa-miR-6887-5p hsa-miR-373-3p hsa-miR-548aj-3p hsa-miR-6874-3p hsa-miR-17-5p hsa-miR-548ah-5p hsa-miR-302b-3p hsa-miR-92b-3p hsa-miR-6077 hsa-miR-922 hsa-miR-4695-3p hsa-miR-562 hsa-miR-5581-5p hsa-miR-513a-5p hsa-miR-6515-3p hsa-miR-6499-3p hsa-miR-519b-3p hsa-miR-4534 hsa-miR-26a-2-3p hsa-miR-526b-3p hsa-miR-6511a-5p hsa-miR-363-3p hsa-miR-

Names	Total	microRNAs
		520a-3p hsa-miR-4708-3p hsa-miR-4748 hsa-miR-3689d hsa-miR-8061 hsa-miR-519c-3p hsa-miR-122b-3p hsa-miR-892b hsa-miR-6805-5p hsa-miR-302e hsa-miR-4753-3p hsa-miR-544b hsa-miR-32-5p hsa-miR-7112-3p hsa-miR-372-3p hsa-miR-30c-5p hsa-miR-3192-3p hsa-miR-30a-5p hsa-miR-3189-3p hsa-miR-6844 hsa-miR-6805-3p hsa-miR-519a-3p hsa-miR-548p hsa-miR-338-5p hsa-miR-5691 hsa-miR-4291 hsa-miR-519d-3p hsa-miR-6806-5p hsa-miR-29b-3p hsa-miR-92a-3p hsa-miR-10523-5p hsa-miR-10393-3p hsa-miR-1468-5p hsa-miR-6733-3p hsa-miR-4796-3p hsa-miR-20b-5p hsa-miR-570-3p hsa-miR-6731-3p hsa-miR-4324 hsa-miR-4297 hsa-miR-29a-3p hsa-miR-520b-3p hsa-miR-106b-5p hsa-miR-6083 hsa-miR-1236-3p hsa-miR-3686 hsa-miR-3663-3p hsa-miR-378a-5p hsa-miR-20a-5p hsa-miR-106a-5p hsa-miR-21-3p hsa-miR-6859-5p hsa-miR-3176 hsa-miR-5697 hsa-miR-6801-5p hsa-miR-4464 hsa-miR-5187-3p hsa-miR-1208 hsa-miR-767-3p hsa-miR-1200 hsa-miR-3116 hsa-miR-548ae-3p hsa-miR-520c-3p hsa-miR-33a-5p hsa-miR-3714 hsa-miR-5195-5p hsa-miR-26a-1-3p hsa-miR-302c-3p hsa-miR-3168 hsa-miR-548j-3p hsa-miR-302a-3p hsa-miR-148b-5p hsa-miR-520d-3p hsa-miR-3121-3p hsa-miR-190a-3p hsa-miR-4760-5p hsa-miR-3654 hsa-miR-4503 hsa-miR-6783-5p hsa-miR-548am-3p hsa-miR-4724-3p hsa-miR-30b-5p hsa-miR-4495 hsa-miR-548aw hsa-miR-3609 hsa-miR-548ah-3p hsa-miR-215-5p hsa-miR-3160-3p hsa-miR-4517 hsa-miR-3133 hsa-miR-3125 hsa-miR-4700-3p hsa-miR-1224-3p hsa-miR-145-3p hsa-miR-29c-3p hsa-miR-3914 hsa-miR-520e-3p hsa-miR-10526-3p hsa-miR-30e-5p hsa-miR-1245b-3p hsa-miR-1279 hsa-miR-93-5p hsa-miR-192-5p hsa-miR-3664-5p hsa-miR-4803 hsa-miR-4772-3p hsa-miR-4251 hsa-miR-6734-3p hsa-miR-5584-3p hsa-miR-1910-3p hsa-miR-548aq-3p hsa-miR-6851-5p hsa-miR-3922-3p hsa-miR-767-5p hsa-miR-6795-5p hsa-miR-548x-3p hsa-miR-11399 hsa-miR-302d-3p hsa-miR-33b-5p.

Table S2. Binding regions between miR-137 and its putative target genes, SRC-1, SRC-2, and SRC-3 (miRmap).

Name	Total	microRNAs
SRC-1; SRC-2; SRC-3	62	hsa-miR-3690 hsa-miR-6499-3p hsa-miR-4659a-3p hsa-miR-4668-5p hsa-miR-661 hsa-miR-499b-3p hsa-miR-4525 hsa-miR-330-3p hsa-let-7g-3p hsa-miR-875-3p hsa-miR-320e hsa-miR-3153 hsa-miR-548d-3p hsa-miR-4725-3p hsa-miR-548h-3p hsa-miR-6128 hsa-miR-5586-5p hsa-miR-579 hsa-miR-605 hsa-miR-6514-5p hsa-miR-548ae hsa-miR-4802-5p hsa-miR-5696 hsa-miR-371a-5p hsa-miR-3646 hsa-miR-548ah-3p hsa-miR-145-3p hsa-miR-5010-5p hsa-miR-373-5p hsa-miR-548aq-3p hsa-miR-425-5p hsa-miR-548ac hsa-miR-625-5p hsa-miR-548x-3p hsa-miR-6514-3p hsa-miR-1254 hsa-miR-548aj-3p hsa-miR-5698 hsa-miR-29b-2-5p hsa-miR-493-5p hsa-miR-616-5p hsa-miR-664b-3p hsa-miR-4659b-3p hsa-miR-489 hsa-miR-3148 hsa-miR-3944-5p hsa-miR-143-5p hsa-miR-3116 hsa-miR-371b-5p hsa-miR-4668-3p hsa-miR-3913-3p hsa-let-7a-2-3p hsa-miR-4271 hsa-miR-4254 hsa-miR-548am-3p hsa-miR-105-5p hsa-miR-499a-3p hsa-miR-548z hsa-miR-137 hsa-miR-6124 hsa-miR-4723-5p hsa-miR-3671
SRC-1; SRC-2	624	hsa-miR-576-3p hsa-miR-2682-5p hsa-miR-522-5p hsa-miR-557 hsa-miR-4743-3p hsa-miR-548av-5p hsa-miR-518d-5p hsa-miR-637 hsa-miR-34b-5p hsa-miR-381-3p hsa-miR-4709-3p hsa-miR-374b-3p hsa-miR-17-5p hsa-miR-4674 hsa-miR-448 hsa-miR-515-5p hsa-miR-92b-3p hsa-miR-3140-5p hsa-miR-3940-5p hsa-miR-3680-5p hsa-miR-103a-2-5p hsa-miR-3155a hsa-miR-3977 hsa-miR-377-5p hsa-miR-562 hsa-miR-4328 hsa-miR-6515-3p hsa-miR-3681-3p hsa-miR-520g hsa-miR-5692a hsa-miR-138-1-3p hsa-miR-513b hsa-miR-675-3p hsa-miR-2110 hsa-miR-487a hsa-miR-4486 hsa-miR-2278 hsa-miR-892c-5p hsa-miR-5002-5p hsa-miR-19b-1-5p hsa-miR-26a-2-3p hsa-miR-4677-3p hsa-miR-548ar-3p hsa-miR-202-5p hsa-miR-6511a-5p hsa-miR-548b-5p hsa-miR-4513 hsa-miR-518e-5p hsa-miR-205-5p hsa-miR-496 hsa-miR-6515-5p hsa-miR-6504-5p hsa-miR-4722-5p hsa-miR-4477a hsa-miR-1277-5p hsa-miR-3618 hsa-miR-513a-3p hsa-miR-4799-5p hsa-miR-4735-3p hsa-miR-223-3p hsa-miR-507 hsa-miR-5583-5p hsa-miR-3171 hsa-miR-3908 hsa-miR-186-3p hsa-miR-4257 hsa-miR-10a-3p hsa-miR-5584-5p hsa-miR-6088 hsa-miR-3202 hsa-miR-3149 hsa-miR-551b-5p hsa-miR-603 hsa-miR-4505 hsa-miR-32-5p hsa-miR-5706 hsa-miR-1273f hsa-miR-4777-5p hsa-miR-128 hsa-miR-3074-3p hsa-miR-23a-5p hsa-miR-298 hsa-miR-4326 hsa-miR-3612 hsa-miR-5680 hsa-miR-3682-3p hsa-miR-1207-5p hsa-miR-3189-3p hsa-miR-4438 hsa-miR-939-3p hsa-miR-5003-3p hsa-miR-129-1-3p hsa-miR-4268 hsa-miR-586 hsa-miR-3692-3p hsa-miR-338-5p hsa-miR-4419b hsa-miR-211-3p hsa-miR-200c-3p hsa-miR-6504-3p hsa-miR-4282 hsa-miR-106a-3p hsa-miR-4744 hsa-miR-4652-3p hsa-miR-410 hsa-miR-196a-3p hsa-miR-1285-3p hsa-miR-525-5p hsa-miR-494 hsa-miR-1343 hsa-miR-548az-5p hsa-miR-4690-5p hsa-miR-4717-5p hsa-miR-3678-3p hsa-miR-20b-5p hsa-miR-5692c hsa-miR-1 hsa-miR-4457 hsa-miR-125b-2-3p hsa-miR-3680-3p hsa-miR-548am-5p hsa-miR-2392 hsa-miR-548t-3p hsa-miR-4418 hsa-miR-183-3p hsa-miR-2276 hsa-miR-548f hsa-miR-3119 hsa-miR-3606-3p hsa-miR-106b-5p hsa-miR-569 hsa-miR-149-3p hsa-miR-573 hsa-miR-1236-3p hsa-miR-4680-3p hsa-miR-3686 hsa-miR-4731-3p hsa-miR-3160-5p hsa-miR-378a-5p hsa-miR-519e-5p hsa-miR-20a-5p hsa-miR-106a-5p hsa-miR-2117 hsa-miR-590-3p hsa-miR-5697 hsa-miR-214-5p hsa-miR-4666a-5p hsa-miR-1200 hsa-miR-3199 hsa-miR-4676-3p hsa-miR-2277-3p hsa-miR-3162-5p hsa-miR-4728-5p hsa-miR-4717-3p hsa-miR-518a-5p hsa-miR-509-3p hsa-miR-4496 hsa-miR-4782-5p hsa-miR-4698 hsa-miR-708-3p hsa-miR-33a-5p hsa-miR-4650-5p hsa-miR-1273g-3p hsa-miR-4699-3p hsa-miR-3617-3p hsa-miR-4509 hsa-miR-5004-3p hsa-miR-654-3p hsa-miR-5096 hsa-miR-4679 hsa-miR-429 hsa-miR-508-3p hsa-miR-548d-5p hsa-miR-500b hsa-miR-26a-1-3p hsa-miR-548aa hsa-miR-154-3p hsa-miR-143-3p hsa-miR-4677-5p hsa-miR-

		4299 hsa-miR-5197-5p hsa-miR-519d hsa-miR-5692b hsa-miR-612 hsa-miR-935 hsa-miR-548aq-5p hsa-miR-126-5p hsa-miR-488-5p hsa-miR-3138 hsa-miR-6506-5p hsa-miR-676-3p hsa-miR-3142 hsa-miR-6721-5p hsa-miR-3927-3p hsa-miR-4259 hsa-miR-491-3p hsa-miR-376c-3p hsa-miR-4724-3p hsa-miR-4524a-3p hsa-miR-362-3p hsa-miR-1257 hsa-miR-4667-5p hsa-miR-412 hsa-miR-4798-3p hsa-miR-567 hsa-miR-4728-3p hsa-miR-548q hsa-miR-2052 hsa-miR-4478 hsa-miR-4307 hsa-miR-889 hsa-miR-937-5p hsa-miR-548ap-3p hsa-miR-571 hsa-miR-3175 hsa-miR-4446-5p hsa-miR-4517 hsa-miR-3128 hsa-miR-526a hsa-miR-4709-5p hsa-miR-501-5p hsa-miR-5702 hsa-miR-5580-5p hsa-miR-642a-3p hsa-miR-527 hsa-miR-3185 hsa-miR-4270 hsa-miR-556-5p hsa-miR-4705 hsa-miR-3653 hsa-miR-519a-5p hsa-miR-759 hsa-miR-2116-5p hsa-miR-1915-5p hsa-miR-224-5p hsa-miR-548t-5p hsa-miR-3675-3p hsa-miR-4700-5p hsa-miR-520h hsa-miR-4713-5p hsa-miR-186-5p hsa-miR-339-5p hsa-miR-486-5p hsa-miR-548ay-5p hsa-miR-4320 hsa-miR-4782-3p hsa-miR-1279 hsa-miR-135a-5p hsa-miR-656 hsa-miR-3922-5p hsa-miR-4799-3p hsa-miR-1587 hsa-miR-2053 hsa-miR-93-5p hsa-miR-4452 hsa-miR-548k hsa-miR-764 hsa-miR-518f-5p hsa-miR-4330 hsa-miR-626 hsa-miR-130b-5p hsa-miR-1251 hsa-miR-670 hsa-miR-939-5p hsa-miR-4772-3p hsa-miR-144-5p hsa-miR-379-3p hsa-miR-1304-3p hsa-miR-206 hsa-miR-4715-5p hsa-miR-1972 hsa-miR-340-5p hsa-miR-4272 hsa-miR-219-5p hsa-miR-25-3p hsa-miR-3942-3p hsa-miR-556-3p hsa-miR-548o-5p hsa-miR-367-3p hsa-miR-601 hsa-miR-1298 hsa-miR-5189 hsa-miR-4738-3p hsa-miR-3668 hsa-miR-4434 hsa-miR-509-5p hsa-miR-944 hsa-miR-3184-3p hsa-miR-1265 hsa-miR-3121-5p hsa-miR-3679-3p hsa-miR-140-3p hsa-miR-369-3p hsa-miR-216a-5p hsa-miR-1183 hsa-miR-548h-5p hsa-miR-662 hsa-miR-4736 hsa-miR-3652 hsa-miR-520d-5p hsa-miR-4428 hsa-miR-3147 hsa-miR-4653-3p hsa-miR-27a-3p hsa-miR-3179 hsa-miR-5007-5p hsa-miR-1285-5p hsa-miR-539-5p hsa-miR-3124-3p hsa-miR-33b-5p hsa-miR-4275 hsa-miR-510 hsa-miR-3064-5p hsa-miR-509-3-5p hsa-miR-365b-5p hsa-miR-873-5p hsa-miR-3180-5p hsa-miR-4477b hsa-miR-548ah-5p hsa-miR-7-2-3p hsa-miR-222-5p hsa-miR-3123 hsa-miR-4768-5p hsa-miR-16-1-3p hsa-miR-4777-3p hsa-miR-181a-2-3p hsa-miR-335-3p hsa-miR-4789-3p hsa-miR-219-1-3p hsa-miR-27a-5p hsa-miR-513a-5p hsa-miR-875-5p hsa-miR-1245a hsa-miR-650 hsa-miR-517-5p hsa-miR-4635 hsa-miR-3163 hsa-miR-18b-5p hsa-miR-18a-5p hsa-miR-9-5p hsa-miR-216a-3p hsa-miR-7-1-3p hsa-miR-892c-3p hsa-miR-6507-3p hsa-miR-374a-5p hsa-miR-3662 hsa-miR-4278 hsa-miR-604 hsa-miR-28-3p hsa-miR-3611 hsa-miR-4456 hsa-miR-196a-5p hsa-miR-514b-5p hsa-miR-3607-3p hsa-miR-3146 hsa-miR-4325 hsa-miR-3613-3p hsa-miR-548a-5p hsa-miR-526b-3p hsa-miR-647 hsa-miR-559 hsa-miR-3976 hsa-miR-642b-3p hsa-miR-363-3p hsa-miR-548x-5p hsa-miR-4685-5p hsa-miR-3978 hsa-miR-5683 hsa-miR-3934-5p hsa-miR-6508-5p hsa-miR-4443 hsa-miR-22-5p hsa-miR-3190-3p hsa-miR-548m hsa-miR-182-3p hsa-miR-3173-3p hsa-miR-4448 hsa-miR-4430 hsa-miR-365a-5p hsa-miR-129-5p hsa-miR-380-3p hsa-miR-548n hsa-miR-4733-5p hsa-miR-217 hsa-miR-3942-5p hsa-miR-1245b-5p hsa-miR-548ak hsa-miR-5588-5p hsa-miR-3191-5p hsa-miR-5688 hsa-miR-3679-5p hsa-miR-411-3p hsa-miR-548c-5p hsa-miR-514a-3p hsa-miR-4672 hsa-miR-4753-3p hsa-miR-3925-5p hsa-miR-4697-3p hsa-miR-196b-5p hsa-miR-4699-5p hsa-miR-5787 hsa-miR-5585-3p hsa-miR-4760-3p hsa-miR-619 hsa-miR-203a hsa-miR-195-3p hsa-miR-548aj-5p hsa-miR-548ap-5p hsa-miR-216b hsa-miR-6510-5p hsa-miR-3605-5p hsa-miR-506-5p hsa-miR-299-5p hsa-miR-548p hsa-miR-4470 hsa-miR-5691 hsa-miR-524-5p hsa-miR-4794 hsa-miR-449c-3p hsa-miR-10b-3p hsa-miR-548ay-3p hsa-miR-5011-5p hsa-miR-5100 hsa-miR-200b-3p hsa-miR-548ar-5p hsa-miR-337-3p hsa-miR-1305 hsa-miR-92a-3p hsa-miR-4763-3p hsa-miR-548i hsa-miR-660-5p hsa-miR-6089 hsa-miR-550b-3p hsa-miR-520a-5p hsa-miR-378g hsa-miR-4533 hsa-miR-29a-5p hsa-miR-3155b hsa-miR-3941 hsa-miR-329 hsa-miR-26b-3p hsa-miR-153 hsa-miR-523-5p hsa-miR-4796-3p hsa-miR-

		4262 hsa-miR-4691-5p hsa-miR-4482-3p hsa-miR-5571-5p hsa-miR-6502-5p hsa-miR-4762-3p hsa-miR-129-2-3p hsa-miR-1207-3p hsa-miR-2115-3p hsa-miR-362-5p hsa-miR-1286 hsa-miR-6083 hsa-miR-3675-5p hsa-miR-484 hsa-miR-548as-5p hsa-miR-382-3p hsa-miR-204-3p hsa-miR-4422 hsa-miR-5093 hsa-miR-4441 hsa-miR-4309 hsa-miR-3620-5p hsa-miR-4796-5p hsa-miR-502-5p hsa-miR-5187-3p hsa-miR-1208 hsa-miR-19a-5p hsa-miR-1253 hsa-miR-1233-3p hsa-miR-1185-5p hsa-miR-1273d hsa-miR-149-5p hsa-miR-3198 hsa-miR-4646-5p hsa-miR-5190 hsa-miR-4712-3p hsa-miR-498 hsa-miR-5681b hsa-miR-4795-3p hsa-miR-374b-5p hsa-miR-548w hsa-miR-4445-5p hsa-miR-4772-5p hsa-miR-4786-3p hsa-miR-3158-3p hsa-miR-4427 hsa-miR-23b-5p hsa-miR-568 hsa-miR-596 hsa-miR-3616-5p hsa-miR-5088 hsa-miR-940 hsa-miR-516b-5p hsa-miR-5703 hsa-miR-184 hsa-miR-449c-5p hsa-miR-135b-5p hsa-miR-125a-3p hsa-miR-548av-3p hsa-miR-3187-5p hsa-miR-3121-3p hsa-miR-450b-5p hsa-miR-452-5p hsa-miR-548j hsa-miR-607 hsa-miR-520c-5p hsa-miR-578 hsa-miR-4766-5p hsa-miR-383 hsa-miR-934 hsa-miR-4742-3p hsa-miR-4495 hsa-miR-3609 hsa-miR-548aw hsa-miR-548a-3p hsa-miR-6126 hsa-miR-3973 hsa-miR-548au-5p hsa-miR-452-3p hsa-miR-4719 hsa-miR-19b-2-5p hsa-miR-221-5p hsa-miR-548at-3p hsa-miR-451b hsa-miR-4670-3p hsa-miR-4720-5p hsa-miR-361-3p hsa-miR-4446-3p hsa-miR-628-5p hsa-miR-4708-5p hsa-miR-3192 hsa-miR-100-3p hsa-miR-3133 hsa-miR-203b-3p hsa-miR-4703-5p hsa-miR-5008-5p hsa-miR-4700-3p hsa-miR-27b-5p hsa-miR-659-3p hsa-miR-3929 hsa-miR-4755-3p hsa-miR-32-3p hsa-miR-4273 hsa-miR-2467-3p hsa-miR-4769-3p hsa-miR-548ab hsa-miR-548y hsa-miR-664a-5p hsa-miR-4279 hsa-miR-335-5p hsa-miR-466 hsa-miR-4425 hsa-miR-16-2-3p hsa-miR-4766-3p hsa-miR-1245b-3p hsa-miR-3165 hsa-miR-1827 hsa-miR-1299 hsa-miR-548c-3p hsa-miR-4639-5p hsa-miR-1264 hsa-miR-297 hsa-miR-5583-3p hsa-miR-548l hsa-miR-1283 hsa-miR-548e hsa-miR-629-3p hsa-miR-655 hsa-miR-377-3p hsa-miR-374c-5p hsa-miR-6165 hsa-miR-3150a-3p hsa-miR-577 hsa-miR-4770 hsa-miR-218-1-3p hsa-miR-3129-3p hsa-miR-5003-5p hsa-miR-4804-5p hsa-miR-376a-5p hsa-miR-300 hsa-miR-6507-5p hsa-miR-495-3p hsa-miR-4775 hsa-miR-4507 hsa-miR-4293 hsa-miR-4516 hsa-miR-4475 hsa-miR-409-3p hsa-miR-627 hsa-miR-4666a-3p hsa-miR-513c-3p hsa-miR-3920 hsa-miR-548az-3p hsa-miR-212-5p hsa-miR-519b-5p hsa-miR-544a hsa-miR-15a-3p hsa-miR-3614-3p hsa-miR-33a-3p hsa-miR-548g-3p hsa-miR-5580-3p hsa-miR-4461 hsa-miR-519c-5p hsa-miR-5008-3p hsa-miR-27b-3p hsa-miR-346 hsa-miR-205-3p hsa-miR-5582-3p hsa-miR-548g-5p hsa-miR-513c-5p hsa-miR-4800-5p hsa-miR-6086 hsa-miR-4801 hsa-miR-548as-3p hsa-miR-613 hsa-miR-514b-3p hsa-miR-1225-3p hsa-miR-512-5p hsa-miR-4643 hsa-miR-4687-5p
SRC-1; SRC-3	13	hsa-miR-4633-3p hsa-miR-938 hsa-miR-301b hsa-miR-301a-3p hsa-miR-4295 hsa-miR-130a-3p hsa-miR-138-5p hsa-miR-3667-3p hsa-miR-6131 hsa-miR-3666 hsa-miR-130b-3p hsa-miR-454-3p hsa-miR-6500-5p
SRC-2; SRC-3	23	hsa-miR-4534 hsa-miR-4302 hsa-miR-302a-5p hsa-miR-370 hsa-miR-1246 hsa-miR-582-5p hsa-miR-4804-3p hsa-miR-4720-3p hsa-miR-3658 hsa-miR-3663-5p hsa-miR-5195-3p hsa-miR-376a-2-5p hsa-miR-3134 hsa-miR-432-3p hsa-miR-3145-3p hsa-miR-1178-3p hsa-miR-34c-3p hsa-miR-767-3p hsa-miR-616-3p hsa-miR-4716-3p hsa-miR-145-5p hsa-miR-4780 hsa-miR-3935
SRC-1	242	hsa-miR-665 hsa-miR-5089-3p hsa-miR-5089-5p hsa-miR-202-3p hsa-miR-615-5p hsa-miR-193a-5p hsa-miR-4474-3p hsa-miR-3688-5p hsa-miR-34a-3p hsa-miR-3122 hsa-miR-4274 hsa-miR-101-5p hsa-miR-1248 hsa-miR-6132 hsa-miR-5186 hsa-miR-1238-5p hsa-miR-3074-5p hsa-miR-449a hsa-miR-5001-5p hsa-miR-4765 hsa-miR-4710 hsa-miR-943 hsa-miR-191-3p hsa-miR-5590-5p hsa-miR-19a-3p hsa-miR-1915-3p hsa-miR-3938 hsa-miR-4491 hsa-miR-584-5p hsa-let-7b-5p hsa-miR-19b-3p hsa-miR-302b-5p hsa-miR-1287 hsa-miR-1252 hsa-miR-3135b hsa-miR-4436b-3p hsa-miR-4664-5p hsa-miR-1296 hsa-miR-766-5p hsa-miR-96-

		5p hsa-miR-5579-5p hsa-miR-4721 hsa-miR-17-3p hsa-miR-4286 hsa-miR-3200-3p hsa-miR-641 hsa-miR-1193 hsa-miR-4312 hsa-miR-92a-1-5p hsa-miR-376a-3p hsa-miR-5000-5p hsa-miR-4297 hsa-miR-3676-5p hsa-miR-4758-5p hsa-miR-1255b-5p hsa-let-7g-5p hsa-miR-3655 hsa-miR-765 hsa-miR-4304 hsa-miR-296-5p hsa-miR-2355-3p hsa-miR-148b-3p hsa-miR-4682 hsa-miR-3692-5p hsa-miR-4519 hsa-miR-3661 hsa-miR-4632-5p hsa-miR-1291 hsa-miR-141-5p hsa-miR-323b-5p hsa-miR-608 hsa-miR-4277 hsa-let-7i-5p hsa-miR-584-3p hsa-miR-769-3p hsa-miR-4323 hsa-miR-4665-5p hsa-miR-3129-5p hsa-miR-3960 hsa-miR-4303 hsa-miR-762 hsa-miR-150-3p hsa-miR-4420 hsa-miR-376b-3p hsa-miR-4659a-5p hsa-miR-4778-3p hsa-miR-152 hsa-miR-4261 hsa-miR-5687 hsa-miR-4758-3p hsa-miR-1263 hsa-miR-4659b-5p hsa-miR-1914-5p hsa-miR-3197 hsa-miR-181b-3p hsa-miR-5047 hsa-miR-492 hsa-miR-3158-5p hsa-miR-4742-5p hsa-miR-1237-3p hsa-miR-4476 hsa-miR-4498 hsa-miR-342-5p hsa-miR-4741 hsa-let-7f-5p hsa-let-7d-5p hsa-miR-3616-3p hsa-miR-4514 hsa-miR-1271-5p hsa-let-7c hsa-miR-3936 hsa-miR-3117-5p hsa-miR-4656 hsa-miR-1236-5p hsa-miR-6509-3p hsa-miR-548au-3p hsa-miR-4424 hsa-miR-3688-3p hsa-miR-5191 hsa-miR-876-3p hsa-miR-3922-3p hsa-miR-3673 hsa-miR-4436b-5p hsa-miR-5694 hsa-miR-3659 hsa-miR-3166 hsa-miR-132-3p hsa-miR-3190-5p hsa-miR-4694-3p hsa-miR-1275 hsa-miR-22-3p hsa-miR-1205 hsa-miR-5187-5p hsa-miR-4646-3p hsa-miR-138-2-3p hsa-miR-490-5p hsa-miR-34c-5p hsa-miR-522-3p hsa-miR-4755-5p hsa-miR-3127-5p hsa-miR-6503-3p hsa-miR-542-3p hsa-miR-4284 hsa-miR-5581-5p hsa-miR-4269 hsa-miR-1184 hsa-miR-4797-5p hsa-miR-1301 hsa-miR-3943 hsa-miR-3617-5p hsa-miR-3907 hsa-miR-493-3p hsa-miR-890 hsa-miR-4669 hsa-miR-4726-3p hsa-miR-127-5p hsa-miR-3154 hsa-miR-4675 hsa-miR-24-3p hsa-miR-1238-3p hsa-miR-6505-5p hsa-miR-3136-3p hsa-miR-658 hsa-miR-182-5p hsa-miR-659-5p hsa-miR-4439 hsa-miR-4458 hsa-miR-1909-3p hsa-miR-3909 hsa-miR-4504 hsa-miR-4761-3p hsa-miR-4747-3p hsa-miR-6074 hsa-miR-4637 hsa-miR-5581-3p hsa-miR-6513-5p hsa-miR-6715b-5p hsa-miR-4483 hsa-miR-3657 hsa-miR-148a-3p hsa-miR-4492 hsa-miR-3126-3p hsa-miR-5001-3p hsa-miR-98-5p hsa-miR-4752 hsa-miR-302d-5p hsa-miR-432-5p hsa-miR-1324 hsa-miR-526b-5p hsa-miR-3176 hsa-miR-215 hsa-miR-4753-5p hsa-miR-4764-5p hsa-miR-936 hsa-miR-6501-3p hsa-miR-449b-5p hsa-miR-4636 hsa-miR-212-3p hsa-miR-3913-5p hsa-miR-3189-5p hsa-miR-4468 hsa-miR-93-3p hsa-miR-450b-3p hsa-miR-224-3p hsa-miR-3182 hsa-miR-4792 hsa-miR-4683 hsa-miR-4790-3p hsa-miR-299-3p hsa-miR-363-5p hsa-miR-25-5p hsa-miR-4651 hsa-miR-6722-3p hsa-miR-1255a hsa-miR-4727-5p hsa-miR-5684 hsa-let-7e-5p hsa-miR-4298 hsa-miR-323a-5p hsa-miR-640 hsa-miR-631 hsa-miR-199a-3p hsa-miR-4500 hsa-miR-34a-5p hsa-miR-5695 hsa-miR-192-5p hsa-let-7a-5p hsa-miR-3136-5p hsa-miR-5006-3p hsa-miR-4771 hsa-miR-199b-3p hsa-miR-4639-3p hsa-miR-4658 hsa-miR-574-5p hsa-miR-4692 hsa-miR-4657 hsa-miR-296-3p hsa-miR-877-3p hsa-miR-6087 hsa-miR-1302 hsa-miR-1293 hsa-miR-4502
SRC-2	467	hsa-miR-5197-3p hsa-miR-4493 hsa-miR-3200-5p hsa-miR-2909 hsa-miR-3681-5p hsa-miR-548s hsa-miR-3187-3p hsa-miR-758-5p hsa-miR-4314 hsa-miR-2114-3p hsa-miR-3120-3p hsa-miR-1306-5p hsa-miR-4641 hsa-miR-23a-3p hsa-miR-4520b-3p hsa-miR-4732-5p hsa-miR-4421 hsa-miR-563 hsa-miR-1179 hsa-let-7a-3p hsa-miR-3672 hsa-miR-380-5p hsa-miR-3691-5p hsa-miR-532-3p hsa-miR-4747-5p hsa-miR-4465 hsa-miR-4797-3p hsa-miR-4690-3p hsa-miR-4684-5p hsa-miR-4650-3p hsa-miR-4671-5p hsa-miR-4463 hsa-miR-516a-3p hsa-miR-135a-3p hsa-miR-4763-5p hsa-miR-575 hsa-miR-3162-3p hsa-miR-375 hsa-miR-5196-5p hsa-miR-4748 hsa-miR-142-5p hsa-miR-664b-5p hsa-miR-486-3p hsa-miR-302c-5p hsa-miR-766-3p hsa-miR-4761-5p hsa-miR-3689d hsa-miR-4264 hsa-miR-5699 hsa-miR-200a-5p hsa-let-7b-3p hsa-miR-3910 hsa-miR-885-5p hsa-miR-4715-3p hsa-miR-4731-5p hsa-miR-769-5p hsa-miR-20a-3p hsa-miR-4694-5p hsa-miR-3677-5p hsa-miR-134 hsa-miR-342-3p hsa-

		miR-6716-3p hsa-miR-5587-5p hsa-miR-542-5p hsa-miR-1231 hsa-miR-4255 hsa-miR-199a-5p hsa-miR-4455 hsa-miR-657 hsa-miR-4689 hsa-miR-136-3p hsa-miR-651 hsa-miR-580 hsa-miR-4739 hsa-miR-576-5p hsa-miR-600 hsa-miR-876-5p hsa-miR-3145-5p hsa-miR-543 hsa-miR-4291 hsa-miR-3689a-5p hsa-miR-1228-3p hsa-miR-4685-3p hsa-miR-3925-3p hsa-miR-5585-5p hsa-miR-30a-3p hsa-miR-5006-5p hsa-miR-4711-3p hsa-miR-3591-3p hsa-miR-4432 hsa-miR-3161 hsa-miR-503-3p hsa-miR-3065-5p hsa-miR-5690 hsa-miR-223-5p hsa-miR-4712-5p hsa-miR-4680-5p hsa-miR-1914-3p hsa-miR-379-5p hsa-miR-4678 hsa-miR-26b-5p hsa-miR-5708 hsa-miR-660-3p hsa-miR-4645-5p hsa-miR-1202 hsa-miR-4511 hsa-miR-197-5p hsa-miR-34b-3p hsa-miR-6715b-3p hsa-miR-21-3p hsa-miR-550b-2-5p hsa-miR-599 hsa-miR-5589-5p hsa-miR-4263 hsa-miR-4791 hsa-miR-4673 hsa-miR-885-3p hsa-miR-495-5p hsa-miR-643 hsa-miR-4735-5p hsa-miR-23c hsa-miR-4539 hsa-miR-4310 hsa-miR-770-5p hsa-miR-4722-3p hsa-miR-1323 hsa-miR-4521 hsa-miR-4292 hsa-miR-384 hsa-miR-154-5p hsa-miR-449b-3p hsa-miR-4253 hsa-miR-1226-3p hsa-miR-2681-5p hsa-miR-23b-3p hsa-miR-3613-5p hsa-miR-4762-5p hsa-miR-200b-5p hsa-miR-98-3p hsa-miR-3713 hsa-miR-5195-5p hsa-miR-3157-5p hsa-miR-4774-3p hsa-miR-122-3p hsa-miR-1270 hsa-miR-3168 hsa-miR-516b-3p hsa-miR-10b-5p hsa-miR-148b-5p hsa-miR-3972 hsa-miR-331-5p hsa-miR-3132 hsa-miR-4760-5p hsa-miR-31-5p hsa-miR-3924 hsa-miR-181d hsa-miR-4503 hsa-miR-4999-3p hsa-miR-497-3p hsa-miR-6508-3p hsa-miR-5193 hsa-miR-139-3p hsa-miR-942 hsa-miR-873-3p hsa-miR-9-3p hsa-miR-2681-3p hsa-miR-6073 hsa-miR-4711-5p hsa-miR-6085 hsa-miR-4459 hsa-miR-3650 hsa-miR-4789-5p hsa-miR-4686 hsa-miR-133b hsa-miR-4524b-5p hsa-miR-139-5p hsa-miR-6081 hsa-miR-4795-5p hsa-miR-301a-5p hsa-miR-4756-5p hsa-miR-451a hsa-miR-548ag hsa-miR-155-3p hsa-miR-3125 hsa-miR-103a-3p hsa-miR-199b-5p hsa-miR-4696 hsa-miR-1229-3p hsa-miR-1284 hsa-miR-1229-5p hsa-miR-4499 hsa-miR-4781-5p hsa-miR-1321 hsa-miR-4305 hsa-miR-5094 hsa-miR-6072 hsa-miR-4745-5p hsa-miR-6079 hsa-miR-548ax hsa-miR-6511b-5p hsa-miR-144-3p hsa-miR-3945 hsa-miR-3607-5p hsa-miR-374a-3p hsa-miR-141-3p hsa-miR-3915 hsa-miR-5007-3p hsa-miR-218-5p hsa-miR-663b hsa-miR-4450 hsa-miR-376c-5p hsa-miR-3064-3p hsa-miR-4803 hsa-miR-4251 hsa-miR-4783-3p hsa-miR-892a hsa-miR-136-5p hsa-miR-3689e hsa-miR-5584-3p hsa-miR-3689f hsa-miR-3120-5p hsa-miR-3691-3p hsa-miR-3183 hsa-miR-632 hsa-miR-4684-3p hsa-miR-1537 hsa-miR-4289 hsa-miR-423-5p hsa-miR-181c-5p hsa-miR-5685 hsa-miR-1272 hsa-miR-499b-5p hsa-miR-200a-3p hsa-miR-4703-3p hsa-miR-29b-1-5p hsa-miR-219-2-3p hsa-miR-4526 hsa-miR-3164 hsa-miR-4317 hsa-miR-155-5p hsa-miR-595 hsa-miR-142-3p hsa-miR-4474-5p hsa-miR-411-5p hsa-miR-570-5p hsa-miR-891b hsa-miR-3606-5p hsa-miR-3529-5p hsa-miR-5700 hsa-miR-3152-5p hsa-miR-3916 hsa-miR-502-3p hsa-miR-4754 hsa-miR-5586-3p hsa-miR-4306 hsa-miR-3689b-5p hsa-miR-3918 hsa-miR-4802-3p hsa-miR-185-5p hsa-miR-888-5p hsa-miR-6077 hsa-miR-548o-3p hsa-miR-624-3p hsa-miR-181c-3p hsa-miR-4256 hsa-miR-133a hsa-miR-3118 hsa-miR-6076 hsa-miR-4531 hsa-miR-1290 hsa-miR-4778-5p hsa-miR-378j hsa-miR-6513-3p hsa-miR-4524a-5p hsa-miR-4640-5p hsa-miR-3140-3p hsa-miR-10a-5p hsa-miR-5009-3p hsa-miR-4723-3p hsa-miR-3928 hsa-miR-4726-5p hsa-miR-490-3p hsa-miR-101-3p hsa-miR-4644 hsa-miR-6716-5p hsa-miR-193b-3p hsa-miR-3620-3p hsa-miR-589-3p hsa-miR-5194 hsa-miR-5590-3p hsa-miR-4528 hsa-miR-2116-3p hsa-miR-4512 hsa-miR-625-3p hsa-miR-323a-3p hsa-miR-3065-3p hsa-miR-892b hsa-miR-3143 hsa-miR-4494 hsa-miR-4714-3p hsa-miR-15b-3p hsa-miR-1825 hsa-miR-181b-5p hsa-miR-4645-3p hsa-miR-6509-5p hsa-miR-508-5p hsa-miR-1911-3p hsa-miR-587 hsa-miR-1303 hsa-miR-5589-3p hsa-miR-4417 hsa-miR-4469 hsa-miR-624-5p hsa-miR-1304-5p hsa-miR-193b-5p hsa-miR-376b-5p hsa-miR-3137 hsa-miR-1185-2-3p hsa-miR-3685 hsa-miR-5579-3p hsa-miR-4451 hsa-miR-1273e hsa-miR-3201 hsa-miR-302f hsa-miR-3660 hsa-

		miR-421 hsa-miR-521 hsa-miR-151a-3p hsa-miR-200c-5p hsa-miR-590-5p hsa-miR-5192 hsa-miR-582-3p hsa-miR-106b-3p hsa-miR-570-3p hsa-miR-2113 hsa-miR-622 hsa-miR-3177-5p hsa-miR-506-3p hsa-miR-4670-5p hsa-miR-31-3p hsa-miR-676-5p hsa-miR-5681a hsa-miR-3974 hsa-miR-505-3p hsa-miR-4701-5p hsa-miR-147a hsa-miR-6724-5p hsa-miR-124-3p hsa-miR-96-3p hsa-miR-4529-5p hsa-miR-3184-5p hsa-miR-4633-5p hsa-miR-4676-5p hsa-miR-4290 hsa-miR-3926 hsa-miR-4688 hsa-miR-4464 hsa-miR-1297 hsa-miR-664a-3p hsa-miR-4522 hsa-miR-921 hsa-miR-193a-3p hsa-miR-4529-3p hsa-miR-4776-3p hsa-miR-1243 hsa-miR-30e-3p hsa-miR-20b-3p hsa-miR-4481 hsa-miR-5188 hsa-miR-6719-3p hsa-miR-4737 hsa-miR-4318 hsa-miR-1262 hsa-miR-2115-5p hsa-miR-4662a-5p hsa-miR-4471 hsa-miR-561-5p hsa-miR-1224-5p hsa-miR-802 hsa-miR-592 hsa-miR-3714 hsa-miR-545-5p hsa-miR-4773 hsa-miR-4653-5p hsa-miR-588 hsa-miR-545-3p hsa-miR-1261 hsa-miR-146a-3p hsa-miR-5087 hsa-miR-5701 hsa-miR-4704-5p hsa-miR-3654 hsa-miR-3152-3p hsa-miR-3174 hsa-miR-1206 hsa-miR-548b-3p hsa-miR-194-5p hsa-miR-1322 hsa-miR-148a-5p hsa-miR-609 hsa-miR-4716-5p hsa-miR-4440 hsa-miR-620 hsa-miR-4329 hsa-let-7f-2-3p hsa-miR-3167 hsa-miR-4660 hsa-miR-2964a-3p hsa-miR-1292-5p hsa-miR-550a-3p hsa-miR-3144-3p hsa-miR-150-5p hsa-let-7f-1-3p hsa-miR-4724-5p hsa-miR-4287 hsa-miR-1227-3p hsa-miR-1256 hsa-miR-3921 hsa-miR-30d-3p hsa-miR-4435 hsa-miR-4266 hsa-miR-3914 hsa-miR-26a-5p hsa-miR-1225-5p hsa-miR-4288 hsa-miR-181a-5p hsa-miR-4662a-3p hsa-miR-3674 hsa-miR-4308 hsa-miR-4701-3p hsa-miR-374c-3p hsa-miR-4729 hsa-miR-7-5p hsa-miR-4433-3p hsa-miR-5689 hsa-miR-130a-5p hsa-miR-1278 hsa-miR-382-5p hsa-miR-888-3p hsa-miR-3115 hsa-miR-107 hsa-miR-5010-3p hsa-miR-4733-3p hsa-miR-3940-3p hsa-miR-6071 hsa-miR-218-2-3p hsa-miR-183-5p hsa-miR-135b-3p hsa-miR-548ai hsa-miR-501-3p hsa-miR-548ao-5p hsa-miR-1226-5p hsa-miR-103b hsa-miR-3665 hsa-miR-5095 hsa-miR-4767 hsa-miR-4423-3p hsa-miR-21-5p hsa-miR-649 hsa-miR-4786-5p hsa-miR-1185-1-3p hsa-miR-433 hsa-miR-3529-3p hsa-miR-4252 hsa-miR-4768-3p hsa-miR-3159 hsa-miR-1976
SRC-3	29	hsa-miR-140-5p hsa-miR-500a-5p hsa-miR-320c hsa-miR-6718-5p hsa-miR-3670 hsa-miR-874 hsa-miR-323b-3p hsa-miR-194-3p hsa-miR-532-5p hsa-miR-3157-3p hsa-miR-1910 hsa-miR-4662b hsa-miR-4429 hsa-miR-597 hsa-miR-455-3p hsa-miR-203b-5p hsa-miR-4426 hsa-miR-1273g-5p hsa-miR-5571-3p hsa-miR-320d hsa-miR-1277-3p hsa-miR-320a hsa-miR-4647 hsa-miR-320b hsa-miR-6080 hsa-miR-4473 hsa-miR-1234-5p hsa-miR-4671-3p hsa-miR-5693

Table S4. Overview of Intersections: Platforms TargetScan, miRDB, and miRmap.

Name	Total	microRNAs
TargetScan; miRDB; miRmap	2	hsa-miR-137 hsa-miR-3646
TargetScan; miRDB;	9	hsa-miR-548t-5p hsa-miR-548c-3p hsa-miR-4699-3p hsa-miR-3163 hsa-miR-1283 hsa-miR-8485 hsa-miR-548az-5p hsa-miR-1277-5p hsa-miR-3191-5p
TargetScan; miRmap	24	hsa-miR-145-3p hsa-miR-3690 hsa-miR-548aj-3p hsa-miR-875-3p hsa-miR-4659b-3p hsa-miR-3913-3p hsa-let-7a-2-3p hsa-miR-4659a-3p hsa-miR-4271 hsa-miR-29b-2-5p hsa-miR-4254 hsa-miR-4725-3p hsa-miR-3148 hsa-miR-548am-3p hsa-miR-548aq-3p hsa-miR-4668-5p hsa-miR-493-5p hsa-miR-548ah-3p hsa-miR-548x-3p hsa-miR-6128 hsa-miR-6124 hsa-miR-143-5p hsa-let-7g-3p hsa-miR-3671
miRDB; miRmap	0	
TargetScan	173	hsa-miR-557 hsa-miR-4743-3p hsa-miR-329-3p hsa-miR-5787 hsa-miR-548ae-3p hsa-miR-4728-5p hsa-miR-23c hsa-miR-6750-3p hsa-miR-195-3p hsa-miR-365b-5p hsa-miR-335-5p hsa-miR-513b-5p hsa-miR-16-2-3p hsa-miR-548aj-5p hsa-miR-548ah-5p hsa-miR-7-2-3p hsa-miR-4766-3p hsa-miR-1245b-3p hsa-miR-4795-3p hsa-miR-6891-5p hsa-miR-506-5p hsa-miR-708-3p hsa-miR-374b-5p hsa-miR-3123 hsa-miR-1299 hsa-miR-33a-5p hsa-miR-4268 hsa-miR-4427 hsa-miR-4509 hsa-miR-338-5p hsa-miR-181a-2-3p hsa-miR-524-5p hsa-miR-6785-5p hsa-miR-5004-3p hsa-miR-23b-3p hsa-miR-377-5p hsa-miR-562 hsa-miR-429 hsa-miR-513a-5p hsa-miR-4330 hsa-miR-3681-3p hsa-miR-200c-3p hsa-miR-18b-5p hsa-miR-8067 hsa-miR-18a-5p hsa-miR-130b-5p hsa-miR-545-3p hsa-miR-200b-3p hsa-miR-6780b-5p hsa-miR-9-5p hsa-miR-4282 hsa-miR-548aa hsa-miR-23a-3p hsa-miR-489-3p hsa-miR-216a-3p hsa-miR-548j-3p hsa-miR-374c-5p hsa-miR-7-1-3p hsa-miR-130a-5p hsa-miR-651-3p hsa-miR-4652-3p hsa-miR-2110 hsa-miR-1250-3p hsa-miR-3187-5p hsa-miR-29a-5p hsa-miR-374a-5p hsa-miR-1285-3p hsa-miR-3121-3p hsa-miR-3662 hsa-miR-3941 hsa-miR-548f-5p hsa-miR-5692b hsa-miR-26b-3p hsa-miR-612 hsa-miR-6888-5p hsa-miR-514b-5p hsa-miR-4796-3p hsa-miR-7703 hsa-miR-7851-3p hsa-miR-607 hsa-miR-340-5p hsa-miR-3607-3p hsa-miR-126-5p hsa-miR-6507-5p hsa-miR-488-5p hsa-miR-6860 hsa-miR-578 hsa-miR-495-3p hsa-miR-4775 hsa-miR-4766-5p hsa-miR-5692c hsa-miR-3942-3p hsa-miR-3653-3p " hsa-miR-4482-3p hsa-miR-3613-3p hsa-miR-6502-5p hsa-miR-4762-3p hsa-miR-1910-3p hsa-miR-152-5p hsa-miR-6511a-5p hsa-miR-4666a-3p hsa-miR-3920 hsa-miR-1207-3p hsa-miR-362-3p hsa-miR-205-5p hsa-miR-548t-3p hsa-miR-548aw hsa-miR-3609 hsa-miR-597-3p hsa-miR-4477a hsa-miR-548x-5p hsa-miR-362-5p hsa-miR-3679-3p hsa-miR-1468-3p hsa-miR-5580-3p hsa-miR-7850-5p hsa-miR-4719 hsa-miR-6802-3p hsa-miR-382-3p hsa-miR-149-3p hsa-miR-369-3p hsa-miR-1236-3p hsa-miR-3934-5p hsa-miR-655-3p hsa-miR-4735-3p hsa-miR-6508-5p hsa-

		miR-655-5p hsa-miR-4680-3p hsa-miR-410-3p hsa-miR-27b-3p hsa-miR-6885-3p hsa-miR-507 hsa-miR-548m hsa-miR-182-3p hsa-miR-128-3p hsa-miR-548g-5p hsa-miR-513c-5p hsa-miR-3173-3p hsa-miR-6086 hsa-miR-4448 hsa-miR-548ap-3p hsa-miR-7856-5p hsa-miR-544a-3p hsa-miR-365a-5p hsa-miR-4446-5p hsa-miR-361-3p hsa-miR-4517 hsa-miR-590-3p hsa-miR-6883-5p hsa-miR-548n hsa-miR-5589-5p hsa-miR-4796-5p hsa-miR-3128 hsa-miR-520d-5p hsa-miR-5189-5p hsa-miR-5187-3p hsa-let-7c-3p hsa-miR-4731-5p hsa-miR-5688 hsa-miR-1301-5p hsa-miR-551b-5p hsa-miR-4653-3p hsa-miR-1252-3p hsa-miR-6809-3p hsa-miR-6875-3p hsa-miR-27a-3p hsa-miR-4753-3p hsa-miR-603 hsa-miR-4505 hsa-miR-3124-3p hsa-miR-33b-5p
miRDB	0	
miRmap	36	hsa-miR-579 hsa-miR-605 hsa-miR-3116 hsa-miR-1254 hsa-miR-6514-5p hsa-miR-5698 hsa-miR-548ae hsa-miR-4802-5p hsa-miR-371b-5p hsa-miR-6499-3p hsa-miR-4668-3p hsa-miR-499a-3p hsa-miR-5696 hsa-miR-548z hsa-miR-320e hsa-miR-3153 hsa-miR-548d-3p hsa-miR-5010-5p hsa-miR-489 hsa-miR-373-5p hsa-miR-548h-3p hsa-miR-371a-5p hsa-miR-425-5p hsa-miR-548ac hsa-miR-661 hsa-miR-499b-3p hsa-miR-625-5p hsa-miR-4525 hsa-miR-330-3p hsa-miR-6514-3p hsa-miR-616-5p hsa-miR-5586-5p hsa-miR-3944-5p hsa-miR-4723-5p hsa-miR-664b-3p hsa-miR-105-5p

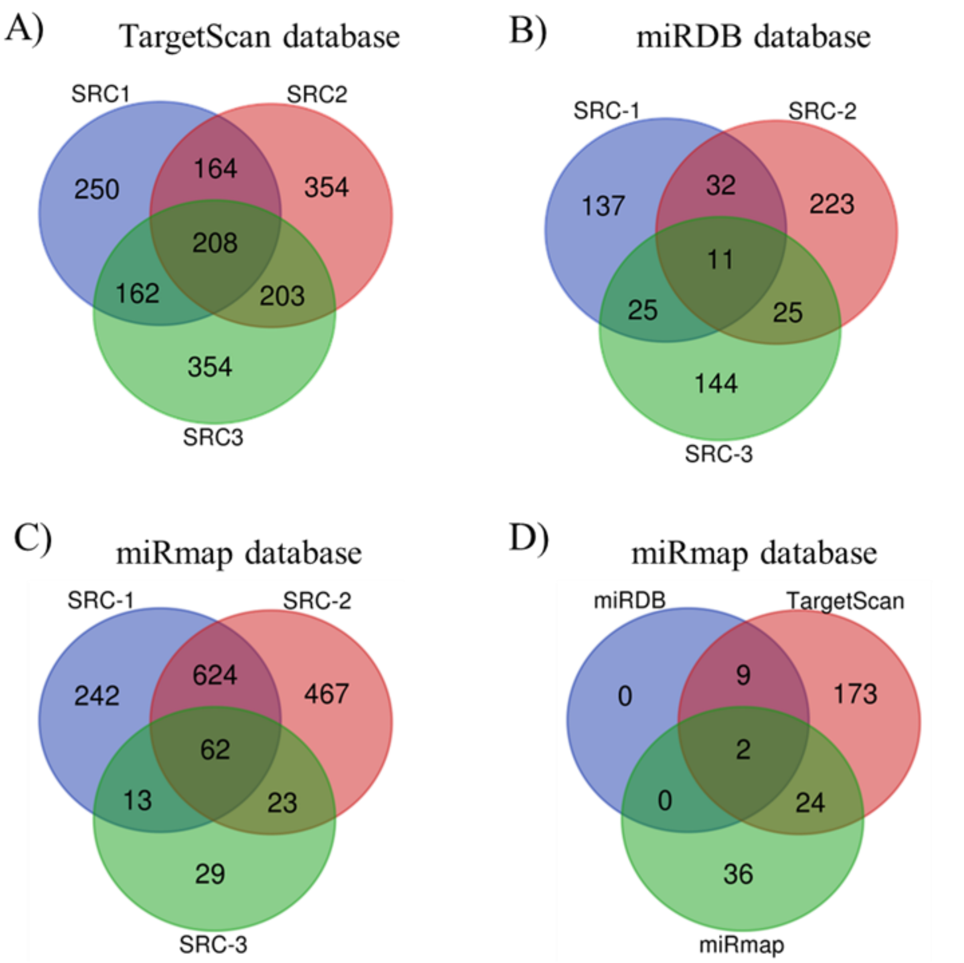

E)

<table>
<tr><th>SRC-1</th><th>Predicted consequential pairing of target region (top) and miRNA (bottom)</th></tr>
<tr><td>Position 862-868 of NCOA1 3' UTR

hsa-miR-137</td><td>5' ...AGUUUGGGGAAAAUGCAAUAAU...
||||||
3' GAUGCGCAUAAGAAUUCGUUAUU</td></tr>
</table>

<table>
<tr><th>SRC-2</th><th>Predicted consequential pairing of target region (top) and miRNA (bottom)</th></tr>
<tr><td>Position 1245-1252 of NCOA2 3' UTR

hsa-miR-137</td><td>5' ...AUUUAUAUGUAAACAAGCAAUAA...
||| |||||||
3' GAUGCGCAUAAGAAU--UCGUUAUU</td></tr>
<tr><td>Position 3553-3559 of NCOA2 3' UTR

hsa-miR-137</td><td>5' ...ACAAGGGUAAAAUUAAGCAAUAU...
|||||||
3' GAUGCGCAUAAGAAUUCGUUAUU</td></tr>
</table>

<table>
<tr><th>SRC-3</th><th>Predicted consequential pairing of target region (top) and miRNA (bottom)</th></tr>
<tr><td>Position 869-876 of NCOA3 3' UTR

hsa-miR-137</td><td>5' ...UUGCCUAUAUGGUAGAGCAAUAA...
||| |||||||
3' GAUGCGCAUAAGAAUUCGUUAUU</td></tr>
<tr><td>Position 1933-1940 of NCOA3 3' UTR

hsa-miR-137</td><td>5' ...UUGGUAUCAUUUUCUAGCAAUAA...
||| |||||||
3' GAUGCGCAUAAGAAUUCGUUAUU</td></tr>
<tr><td>Position 3245-3251 of NCOA3 3' UTR

hsa-miR-137</td><td>5' ...AGAAUAAAAUAUGAG--AGCAAUAU...
||| |||||||
3' GAUGCGCAUAAGAAUUCGUUAUU</td></tr>
</table>

Supplementary Figure S1. The diagrams represent the prediction analysis of microRNA-targets for genes p160 coactivators using three databases: TargetScan, miRDB, and miRmap. A) The TargetScan database displays 208 microRNAs that are common to all three genes (SRC-1, SRC-2, and SRC-3). B) The miRDB database displays 11 microRNAs that are common to all three genes. C) The miRmap database displays 62 microRNAs that are common to all three genes. D) Venn diagram shows the intersection of microRNAs for SRC-1, SRC-2, and SRC-3 genes among the three databases. Among all the microRNAs identified, only hsa-miR-137 and hsa-miR-3646 showed interaction regions with three p160 coactivators. E) Sequence of the binding regions between miR-137 and its targets, SRC-1, SRC-2, and SRC-3.

Eedunuri,VK et al. *Mol Endocrinol*, 2015.

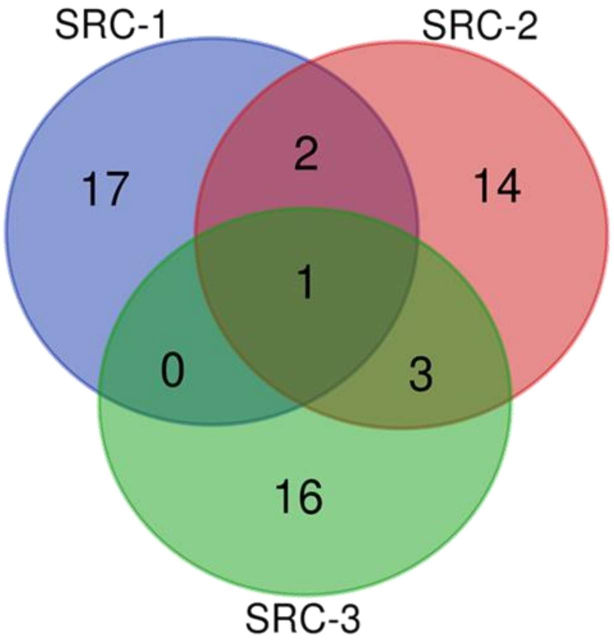

Names	Total	microRNAs
SRC-1; SRC-2; SRC-3	1	miR-137.
SRC-1; SRC-2	2	miR-590-3p; miR-495.
SRC-1; SRC-3	0	-
SRC-2; SRC-3	3	miR-429; miR-200b; miR-200c.
SRC-1	17	miR-18a; miR-219-5p; miR-454; miR-130a; miR-374a; miR-374b; miR-23a; miR-301a; miR-182; miR-32; miR-216a; miR-34a; miR-129-5p; miR-130b; miR-152; miR-18b; miR-301b.
SRC-2	14	miR-381; miR-21; miR-410; miR-448; miR-181b; miR-181c; mIR-186; miR-181a; miR-875-5p; miR-4282; miR-214-5p; miR-590-5p; miR-199a; miR-30.
SRC-3	16	miR-17-5p; miR-520d-3p; miR-106a; miR-20a; miR-216b; miR-519d; miR-29b; miR-372; miR-520e; miR-520c-3p; miR-29c; miR-29a; miR-106b; miR-93; miR-20b; miR-520b.

Supplementary Figure S2. In silico prediction of the microRNA targets for p160 coactivators based on the study validated by Eedunuri et al. (2015).

DTAPEP
Diretoria Técnica de Apoio ao Ensino e Pesquisa

AUTENTICAÇÃO DE LINHAGENS

Laudo do Perfil de STR´s / STR Profile Report

INFORMAÇÃO DO CLIENTE / *CUSTOMER INFORMATION*

**Nome** / *Name*	IRAN AMORIM DA SILVA
**Instituição** / *Institution*	HCFMUSP
**Departamento** / *Department*	CIRURGIA
**Telefone** / *Phone*	1130617183
**E-mail**	iransilva@gmail.com

**Nome da Linhagem** / *Cell line name*	PC3LUC2 (ATCC CRL1435LUC2)

**Amostra recebida em** / *Samples received in* 00-00-0000	**Data do laudo** / *Report date*: 16-12-2019

Loci	Resultado do Teste / *Test Result* (amostra/*sample*)				Perfil da referência no Banco de Dados / *Reference Database Profile*				
	Perfil da Amostra / *Query Profile* PC3LUC2				Perfil da referência / *Database Profile* PC-3 (ATCC:CRL-1435)				
**TH01**	6	7			6	7			
**TPOX**	8	9			8	9			
**vWA**	17				17				
**CSF1PO**	11				11				
**D16S539**	11				11				
**D7S820**	8	11			8	11			
**D13S317**	11				11				
**D5S818**	13				13				
**D21S11**									
**Amelogenin**	x				x				
**Número de alelos iguais entre a amostra e referência** / *Number of shared alleles between query sample and database profile*:									12
**Total de alelos na amostra referência** / *Total number of alleles in the database profile*:									12
**Porcentagem de correspondência entre alelos da amostra e da referência** / *Percent match between the submitted sample and the database profile*:									100

RESULTADO / *RESULT*
Linhagens celulares com uma porcentagem de correspondência >=80% são consideradas relacionadas, ou seja, derivada de um ancestral comum. Linhagens com porcentagem de correspondência entre 55% e 80% necessitam de novos testes (maior número de marcadores) para confirmar sua autenticidade. / *Cell lines with >=80% match are considered to be related; i.e., derived from a common ancestry. Cell lines with between a 55% to 80% match require further profiling for authentication of relatedness. (ATCC - STR Profile Report)*

METODOLOGIA / *METHODOLOGY*
10 STR's, correspondendo aos loci TH01, TPOX, vWA, CSF1PO, D16S539, D7S820, D13S317, D5S818, Amelogenin e D21S11 (GenePrint 10 / Promega), foram amplificados e submetidos à eletroforese capilar para separação dos fragmentos (3730 DNA Analyzer - Applied Biosystems). Os dados foram analisados através do software XXX GeneMaker HDI v.1.1.0 (Softgenetics LCC). Controles positivos e negativos apropriados foram utilizados na validação de cada amostra. / *10 STR's (TH01, TPOX, vWA, CSF1PO, D16S539, D7S820, D13S317, D5S818, Amelogenin and D21S1- GenePrint 10 / Promega) are co-amplified and submitted to capillary electrophoresis (3730 DNA Analyzer - Applied Biosystems). An internal lane standard (ILS) and allelic ladder are provided for sizing and genotyping of amplified fragments, and the 2800M Control DNA is supplied as a positive control (GenePrint 10 / Promega). Data is analyzed using software GeneMaker HDI v.1.1.0 (Softgenetics LCC).*

INTERPRETAÇÃO DOS DADOS / *INTERPRETATION OF DATA*
Os resultados foram interpretados segundo as diretrizes do ANSI Standards 2011 (ASN-0002 / ATCC Standards Development Organization) / *Results were interpreted as described in ANSI Standards 2011 (ASN-0002 / ATCC Standards Development Organization / ATCC STR Profile Report)*

[ ] **A linhagem enviada para análise é de origem humana, mas o seu perfil não corresponde a nenhuma linhagem padrão presente nos bancos de dados analisados** / *The submitted sample profile is of human origin, but not a match for any profile in the STR database*

[X] **A linhagem enviada para análise corresponde exatamente à seguinte linhagem celular humana** / *The submitted profile is an exact match for the following human cell lines):*
PC-3 Prostate Adenocarcinoma Human (ATCC:CRL-1435)

[ ] **O perfil de marcadores da linhagem enviada é similar à(s) seguinte(s) linhagem(s) celular(es) humana(s)** / *The submitted profile is similar to the following ATCC human cell line(s):*

OBSERVAÇÕES / *Additional Comments*
https://www.atcc.org/en/STR_Database.aspx Obs: A linhagem parece ter sido geneticamente modificada

Responsável Técnico: Christian Albert Merkel
Email: c.merkel@fm.usp.br

DTAPEP - Diretoria Técnica de Apoio ao Ensino e Pesquisa
Biotério Central da Faculdade de Medicina da USP
Av. Dr. Arnaldo, 455 - Cerqueira César - São Paulo - CEP 01246-903
Fone 11 55 3061-7412
www.bioterio.fm.usp.br

Supplementary Figure S3. Authenticated cellular by Short Tandem Repeat.

Supplementary Table S5. TaqMan™ Assay tables were used in the study.

**Genes**	**Assay**	**Company**
SRC-1	Hs00186661_m1	Applied Biosystems
SRC-2	Hs00896109_m1	Applied Biosystems
SRC-3	Hs00180722_m1	Applied Biosystems
AR	Hs00171172-m1	Applied Biosystems
GAPDH	Hs99999905	Applied Biosystems

Supplementary Table S6. Antibody tables used in work.

**Proteins**	**Cat**	**Company**
SRC-1	128E7	Cell Signaling
SRC-2	D2X4M	Cell Signaling
SRC-3	5E11	Cell Signaling
AR	MA5-13426	Invitrogen
